# Disruption of deoxyribonucleotide triphosphate biosynthesis leads to *RAS* proto-oncogene activation and perturbation of mitochondrial metabolism

**DOI:** 10.1016/j.jbc.2024.108117

**Published:** 2024-12-23

**Authors:** Rodolphe Suspène, Kyle A. Raymond, Pablo Guardado-Calvo, Julien Dairou, Frédéric Bonhomme, Christine Bonenfant, Serge Guyetant, Thierry Lecomte, Jean-Christophe Pagès, Jean-Pierre Vartanian

**Affiliations:** 1 Virus and Cellular Stress Unit, Department of Virology, Université Paris Cité, Institut Pasteur, Paris, France; 2 Sorbonne Université, Complexité du Vivant ED515, Paris, France; 3 Structural Biology of Infectious Diseases, Department of Virology, Université Paris Cité, Institut Pasteur, Paris, France; 4 Université Paris Cité, CNRS, Laboratoire de Chimie et de Biochimie Pharmacologiques et Toxicologiques, Paris, France; 5 Epigenetic Chemical Biology Unit, UMR CNRS 3523, Université Paris Cité, Institut Pasteur, Paris, France; 6 Pathology Department and Cancer Molecular Genetics Platform, CHRU de Tours Hôpital Trousseau, Tours, France; 7 Inserm UMR 1069, N2COx "Niche, Nutrition, Cancer and Oxidative Metabolism", Université de Tours, Tours, France; 8 Service de gastroentérologie, CHRU de Tours Hôpital Trousseau, Tours, France; 9 RESTORE, Université de Toulouse, EFS Occitanie, INP-ENVT, INSERM U1301, UMR CNRS 5070, Toulouse, France; 10 CHU de Toulouse, IFB, Hôpital Purpan, Toulouse, France

**Keywords:** *RAS* gene, nucleotide, DNA, mutation, mitochondria, cancer

## Abstract

Perturbation of the deoxyribonucleotide triphosphate (dNTP) pool is recognized for contributing to the mutagenic processes involved in oncogenesis. The *RAS* gene family encodes well-characterized oncoproteins whose structure and function are among the most frequently altered in several cancers. In this work, we show that fluctuation of the dNTP pool induces CG → TA mutations across the whole genome, including *RAS* gene at codons for glycine 12 and 13, known hotspots in cancers. Cell culture addition of the ribonucleotide reductase inhibitor thymidine increases the mutation frequency in nuclear DNA and leads to disruption of mitochondrial metabolism. Interestingly, this effect is counteracted by the addition of deoxycytidine. Finally, screening for the loss of hydrogen bonds detecting CG → TA transition in *RAS* gene of 135 patients with colorectal cancer confirmed the clinical relevance of this process. All together, these data demonstrate that fluctuation of intracellular dNTP pool alters the nuclear DNA and mitochondrial metabolism.

A balanced concentration of deoxyribonucleotide triphosphates (dNTPs) is crucial for achieving successful nuclear DNA (nuDNA) replication and repair (1). Temporal, spatial, and ratio imbalances of intracellular dNTPs have been linked to mutagenic and cytotoxic effects on cells (2). Therefore, maintaining the balance between dNTP biosynthesis and the degradation processes contribute to cellular genome homeostasis. Multiple oncogenic signaling pathways alter dNTP metabolism, which successively play a conspicuous role in cancer initiation and progression (3, 4).

Cancer development involves several distinct mechanisms, among which unscheduled DNA synthesis or disruptions in dNTP metabolism, contributing to genome instability, a regular feature in tumorigenesis (2, 4, 5). Defects in DNA repair pathways can also exacerbate cancer development through the acquisition of mutations and genetic rearrangements. The path to a transformed phenotype has been associated with disrupted activation of several types of cellular oncogenes. In that respect, the *RAS* family members encode three ubiquitously expressed genes, *HRAS*, *KRAS*, and *NRAS*, sharing significant sequence homology and largely overlapping functions. They are among the oncoproteins whose structure and function were most thoroughly characterized (6, 7). In particular, *HRAS* and *KRAS* proto-oncogenes are good archetypes representing one of the most frequent oncoproteins associated with human cancer (6), with ∼20% of cancers driven by one or multiple point mutations in these genes (8).

The *RAS* superfamily consists of genes encoding small proteins that can switch from an inactive GDP-bound state to an active GTP-bound state. Some mutations lead to continuously active forms triggering downstream signaling of cellular proliferation, invasion, migration, and cytoskeletal organization inducing cell tumorigenesis (9). Accordingly, several single point mutations in mammalian *HRAS* or *KRAS* genes are well characterized in their ability to induce cellular transformation (10, 11). These mutations in *HRAS* or *KRAS* proto-oncogenes have been conspicuously linked to initiation of human malignancies such as pancreatic ductal adenocarcinoma, colorectal carcinoma, multiple myeloma, lung adenocarcinoma, or head and neck cancers (12), a role extensively confirmed and deciphered in different experimentally induced tumors in animal models (6).

Most oncogenic *RAS* mutations are gain of function, clustering in three hotspots at codons *H/KRAS*^*Gly12*^, *H*/*KRAS*^*Gly13*^, and *H/KRAS*^*Gln61*^ (13). Interestingly, while ∼44 distinct point mutations have been characterized in *RAS* isoforms, ∼95% of all mutations occur at codons Gly_12_, Gly_13_, and Gln_61_ (6, 7, 14). These key oncogenic mutations are localized in structurally homologous region between the three isoforms. Of note, glycine residue at position 12 or 13 (*H/KRAS*^*Gly12,13*^) appears to be central in the regulated function of H/KRAS protein. Substitutions at these residues result in constitutive oncogene activation.

The maintenance of dNTP pools is critical for various cellular pathways. It was shown that a balanced pool of dNTPs was essential for high fidelity during DNA replication (15). Biased availability of dNTP levels occurring at the replication site are mutagenic, while experimentally induced imbalances in dNTP pools have been associated with multiple mutations occurring randomly in the genome (16, 17, 18, 19).

In mammalian cell lines, the relative intracellular dNTP concentrations are naturally biased with [dTTP] ≥ [dATP] > [dCTP] ≥ [dGTP], and a [dTTP]/[dCTP] ratio varying between 2- and 10-fold (18). The ribonucleotide reductase (RNR) is an essential multisubunit allosteric enzyme found in all living organisms, catalyzing the rate-limiting step in dNTP synthesis, namely the conversion of ribonucleoside diphosphates to deoxyribonucleoside diphosphates. RNR consist of a large subunit called RR1, which contains two allosteric sites and one catalytic site, and a small subunit called RR2, which houses a tyrosyl free radical essential for initiating catalysis (20). As this is the rate-limiting step in dNTP synthesis, RNR is crucial for maintaining a balanced nucleotide pool within the cell and is integral to DNA synthesis (21). The synthesis of dTTP, comes from both deoxythymidine (dThy), and the scavenging and metabolism of dUMP to dTMP (22). Adding dThy to culture medium leads to an increase in the intracellular [dTTP] concentration through its allosteric effect on RNR. It also activates a negative feedback pathway with the reduction of CDP to dCDP, resulting in a decrease in intracellular [dCTP] concentration (22, 23, 24). Nevertheless, the deleterious effect of dThy can be counteracted by the addition of deoxycytidine (dCyd), which upon phosphorylation will restore the intracellular pool of [dCTP] (25, 26).

Fluctuations of the dNTP pool may also significantly affect mitochondrial functions, as these organelles are involved in a wide range of fundamental cellular processes, including cellular energy production, thermogenesis, lipid biosynthesis, and cell death (27). Indeed, deficient dNTP levels could provoke replication pauses or synthesis errors in both nuDNA and mitochondrial DNA (mtDNA), leading to an impaired mitochondrial network and affect its cellular energy production (28). Optimal mitochondrial function is essential for maintaining a balanced cellular energy supply, and a disruption in the dNTP pool may compromise mitochondrial function (5).

In this work, we investigated fluctuations of the dNTP pools and demonstrate that these fluctuations induce CG → TA mutations scattered throughout the *HRAS* gene. Accordingly, random mutagenesis performed on WT *HRAS*^*Gly12*^ proto-oncogene using biased dNTPs led to select mutated *HRAS* variants and a cell morphology transformation. Furthermore, addition of dThy to cell *in vitro* damaged the mitochondrial network metabolism and increased the frequency of *HRAS*^*Gly12*^ and *HRAS*^*Gly13*^ variants, which was counteracted by the addition of dCyd. Finally, *KRAS*^*Gly12*,^ ^*Gly13*^ mutations were analyzed in 135 patients with colorectal cancer presenting CG → TA transitions. By using a screening method based on the loss of hydrogen bonds following these mutations, we were able to confirm the mutational burdens of these patients.

All together, these data demonstrate that the fluctuation of the intracellular dNTP pool has an impact on nuDNA and mtDNA. The bias in nucleotides leads to random mutations in the *HRAS* and *KRAS* genes, as well as widespread mutations throughout the genome, resulting in impaired mitochondrial metabolism.

## Results

### *HRAS*^*Gly12*^ mutagenesis with biased deoxynucleotides

**Figure 1:**
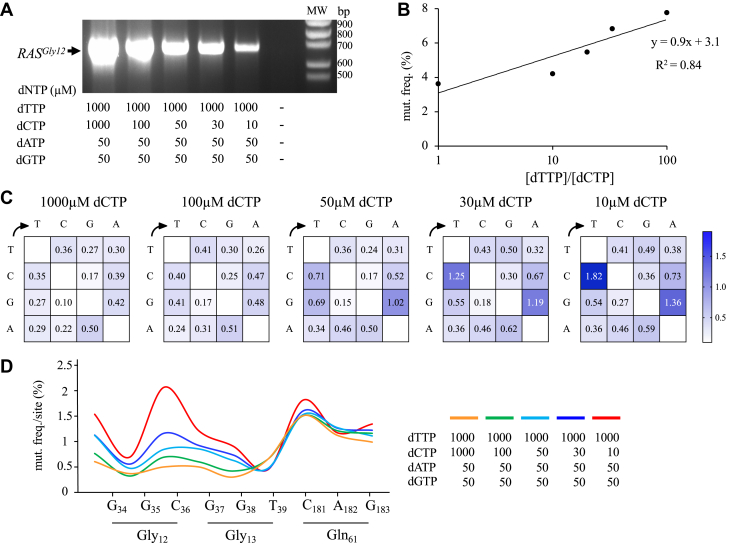
**PCR mutagenesis of *HRAS* cDNA.***A*, agarose gel analysis of the PCR-amplified *HRAS* cDNA reactions. PCR were performed with dTTP: 1000 μM, dATP and dGTP: 50 μM and dCTP: 10 μM; 30 μM, 50 μM, 100 μM, and 1000 μM. *B*, mutation frequency is proportional to the [dTTP]/[dCTP] ratio. Conditions used where those described in A. *C*, heat map representing the mutation matrices for *HRAS* cDNA analyzed from NGS data. Mutagenesis was performed with 10 μM to 1000 μM dCTP. The proportion of CG → TA is proportional to the [dTTP]/[dCTP] ratio. *D*, site specific frequency analysis of editing *HRAS* cDNA recovered by NGS. Mutation frequencies were analyzed for the three hotspots present in *HRAS* gene, Gly_12_, Gly_13_, and Gln_61_ known to be mainly mutated in cancer. The mutation frequency per nucleotide is correlated to the [dTTP]/[dCTP] ratio. cDNA, complementary DNA; MW, molecular weight; NGS, next-generation sequencing.

Mutagenic PCR (mut-PCR) was used to introduce random mutations on the 576 bp corresponding to the complete *HRAS* complementary DNA (cDNA) proto-oncogene (*HRAS*^*Gly12*^). Mut-PCR takes advantage of the inherent low fidelity of the Taq DNA polymerase, which may be further decreased by using unequal dNTP concentrations (29). PCRs were performed with biased dNTPs to obtain a library of the *HRAS*^*Gly12*^ cDNA random mutants. As the majority of *HRAS* or *KRAS* mutations detected in patients are CG → TA mutations (30), we used nucleotide biases to generate such transitions during PCR. Hence, a [dTTP] > [dCTP] nucleotide bias allowed enrichment in A and T during PCR. To ensure a high ratio between these two nucleotides, the concentration of [dTTP] was adjusted to 1000 μM, while the concentration of [dCTP] was varying from 10 μM to 1000 μM, [dATP] and [dGTP] were both maintained at 50 μM. As observed in Figure 1*A*, efficiency of the PCR product decreased as the ratio of [dTTP]/[dCTP] increased, highlighting the clustering of mutations during the PCR process and the difficulty for the Taq polymerase to accurately copy the template.

The mutation frequency was verified by next-generation sequencing (NGS). For each sample, reads obtained by Illumina sequencing were checked for the quality and assessed using FastQC. The relationship between the proportion of mutations with increased [dTTP]/[dCTP] ratio reflects the extent of DNA mutation. The mutation frequency obtained was proportional to the [dTTP]/[dCTP] ratio, with the most restrictive bias yielding values as high as ∼7.7% (Fig. 1*B*).

This frequency of mutated *HRAS*^*Gly12*^ cDNA led to ∼2 to 3 amino acid substitutions per sequence, which were generally well distributed throughout the gene (not shown). As expected, the vast majority of substitutions presented as CG → TA transitions based on the dG:dT mispairing occurring on both DNA strands due to the [dTTP] > [dCTP] bias (Fig. 1*C*). As can be seen from the mutation matrices, the CG → TA mutation frequencies increased as a function of the [dTTP]/[dCTP] ratio used in the mut-PCR (Fig. 1*C*).

Given that the *HRAS*^*Gly12*^, *HRAS*^*Gly13*^, and *HRAS*^*Gln61*^ positions are predominantly involved during oncogenesis, the mutation frequencies located at each specific nucleotide position corresponding to these three amino acids were analysed (Fig. 1*D*). Interestingly, we observed that the mutation frequencies per site increased as a function of the deoxynucleotide bias used. This occurred mainly at nucleotides positioned at G_34_, G_35_, C_36_, G_37_, G_38_, C_181_, and G_183_ which remains consistent with the expected mismatches. As nucleotides T_39_ and A_182_ were not associated with specific mismatches when strong [dTTP]/[dCTP] bias was used, they appeared to be less affected (Fig. 1*D*). Overall, these data highlight the possibility of generating a heterogenous mutant library by mut-PCR correlated to the concentration of dNTPs, leading to CG → TA mutations in the *HRAS* gene.

### Expression of random *HRAS* variants leads to a transformed cellular phenotype

**Figure 2:**
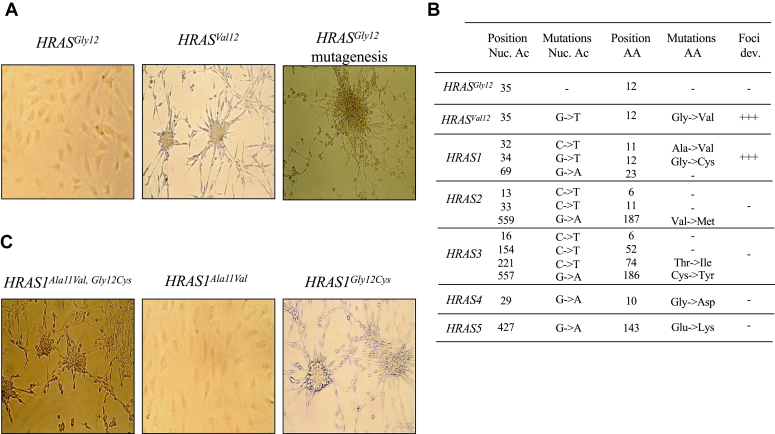
**Cellular phenotype of NIH-3T3 cells following electroporation with *HRAS* cDNA.***A*, *HRAS*^*Gly12*^, *HRAS*^*Val12*^, and *HRAS*^*Gly12*^ mutagenesis corresponding, respectively, to the HRAS proto-oncogene (Gly_12_, WT), oncogene *HRAS* (Val_12_), and *HRAS* mutagenesis were electroporated in NIH-3T3. Cellular transformed phenotypes appeared with *HRAS*^*Val12*^ and *HRAS* mutagenesis. *B*, collection of five *HRAS* (*HRAS1-5*) mutants derived from *HRAS*^*Gly12*^ mutagenesis with a dNTP bias involving a [dTTP] > [dCTP]. *HRAS*^*Gly12*^ and *HRAS*^*Val12*^ were used as negative and positive controls, respectively. The mutations are described according to the type of mutation, as well as the nucleotide and amino acid position. “+” and “−” correspond, respectively, to the presence or absence of phenotypic transformation of the cells. *C*, analysis of each mutation detected in *HRAS1*^*Ala11Val, Gly12Cys*^ double mutant. dNTP, deoxyribonucleotide triphosphate.

To obtain a wide heterogeneous library of *HRAS* mutants, a 100/1 ratio of [dTTP]/[dCTP] was used ([dTTP]: 1000 μM, [dCTP]: 10 μM, [dATP], and [dGTP]: 50 μM). Mut-PCR products were then cloned in a pSV2-gpt expressing vector and electroporated into NIH-3T3 cells. WT proto-oncogene *HRAS*^*Gly12*^ and the mutated *HRAS*^*Val12*^ oncogene were used as negative and positive controls, respectively. After 3 weeks post electroporation, the ability of *HRAS*^*Val12*^ to induce growth transformation of NIH-3T3 cells was clearly observed, whereas *HRAS*^*Gly12*^ showed no growth abnormalities (Fig. 2*A*). As morphological changes develop in the cell culture, numerous visible foci emerge, detaching from the surrounding monolayer of untransformed cells and exhibiting a loss of contact inhibition. These foci are characterized by their dense, often rounded, and more refractive appearance compared to the untransformed cells (Fig. 2, *A* and *B*). Interestingly, the electroporated mutagenic library obtained from the WT *HRAS*^*Gly12*^ also generated transform clones as obtained with *HRAS*^*Val12*^ (Fig. 2*A*).

Investigation of the transformed cells obtained by the mutagenic *HRAS*^*Gly12*^ library revealed upon sequencing, a collection of 5 *HRAS* sequences with mutations scattered through the complete cDNA (*HRAS1*–*HRAS5*, Fig. 2*B*). The distribution showed between 1 and 3 mutations per gene with an equivalent proportion of G → A and C → T transitions (Fig. 2*B*). *HRAS1* possessed 3-point mutations, C_32_ → T, G_34_ → T, and G_69_ → A corresponding respectively to Ala_11_, Gly_12_, and Leu_23_ amino acids. As expected, the Gly_12_ → Cys amino acid change confers the oncogenic properties akin to *HRAS*^*Val12*^. To assess the impact of each mutation located in *HRAS1* (Ala_11_ → Val and Gly_12_ → Cys), directed mutagenesis was performed on *HRAS*^*Gly12*^. The two generated individual variants were then cloned in a pSV2-gpt expression vector. To confirm whether these single *HRAS* mutants could have any role in cellular transformation, they were electroporated into NIH-3T3 cells. After 3 weeks of culture, alterations of the cell morphology were visible and were detected only with the *HRAS*^*Cys12*^ variant (Fig. 2*C*). All nonsynonymous mutations detected in *HRAS2*, *HRAS3*, *HRAS4*, and *HRAS5* have been electroporated separately showing no impact on the cell phenotype transformation (Fig. 2*B*). This indicates that these variants were recovered after selection in culture through coexpression with the transforming Gly_12_ → Cys variant, by co-insertions of these different variants in electroporated cells.

**Figure 3:**
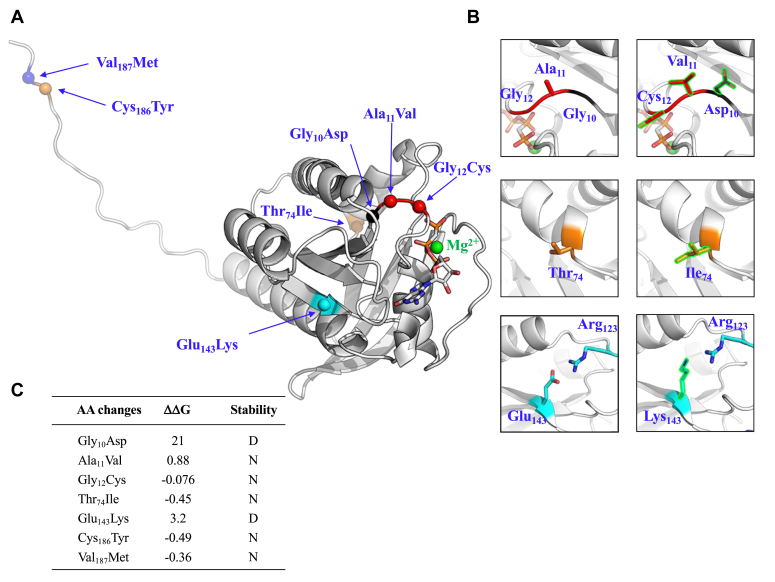
**Localization of mutations on the HRAS crystallographic structure.***A*, HRAS1-5 mutations mapped onto an HRAS model generated using AlphaFold2. The localization of each mutant is represented by a *sphere* and is labeled. *B*, zoomed views of the mutated residues using the same color code as in (*A*) and labeled. The WT residue. *C*, FoldX5 predictions of the free energy changes (ΔΔG) for each *HRAS1*-*5* mutant with respect to the *HRAS*^*Gly12*^ WT protein. Ala_11_Val, Gly_12_Cys, Tyr_74_Ile, Cys_186_Tyr, and Val_187_Met are structurally neutral (N), while Gly_10_Asp and Glu_143_Lys mutations destabilize (D) the protein.

To estimate the effect of the seven single detected mutations (*HRAS 1*–*5*) on the stability of HRAS protein, we estimated the Gibbs free energy changes (ΔΔG) induced by each point mutations using the position Scan method implemented in FoldX-5 (https://foldxsuite.crg.eu/). The crystal structure of HRAS (PDB code: 121P, resolution 1.5 Å) was used to analyze the potential impact of each mutation. In parallel, we performed a 3D modeling of all mutations using PyMOL (http://github.com/schrodinger/pymol-open-source) (Fig. 3, *A* and *B*). As guideline, we considered a mutation as stabilizing if the ΔΔG is < −3 kcal/mol, destabilizing if ΔΔG >3 kcal/mol and neutral if otherwise) (Fig. 3*C*). Mutations in positions Cys_186_ → Tyr (ΔΔG = −0.49) and Val_187_ → Met (ΔΔG = −0.36), which are both located in the disordered C-terminal tail of the crystal structure, are unlikely to significantly impact protein stability. The side chains of the mutated residues at positions Cys_12_ (ΔΔG = −0.076), Val_11_ (ΔΔG = 0.88), and Ile_74_ (ΔΔG = −0.45) are solvent exposed on the protein surface and are not predicted to affect HRAS protein stability (Fig. 3*B*). However, this does not mean they do not influence the protein’s catalytic activity, as evidenced by the Gly_12_ → Cys mutation. Notably, the Glu_143_ → Lys modification is destabilizing (ΔΔG = 3.2) as it disrupts the salt bridge formed between Glu_143_ and Arg_123_. Also, the Gly_10_ → Asp mutation is strongly destabilizing (ΔΔG = 21) because the Asp_10_ side chain projects into the hydrophobic core of the protein (Fig. 3*B*). Overall, the structural analysis is in agreement with the functional assays, suggesting that only one modification (Gly_12_ → Cys) results in enhanced catalytic activity, while two (Glu_143_ → Lys and Gly_10_ → better arrow, same as above Asp) are destabilizing.

### Thymidine-treated cells have a perturbed intracellular dNTP pool with increased *HRAS*^*Gly12*^ mutation frequency

The *RAS* gene family mutated at Gly_12_ and Gly_13_ in ∼35 to 45% of colorectal cancers, leading to insensitive GTPase, is considered as a mutational hotspot (6, 7, 14). To evaluate whether biased dNTPs increase the *HRAS*^*Gly12*^ and/or *HRAS*^*Gly13*^ mutation frequencies, HeLa cells were incubated for 24 h with 10 μM or 30 μM thymidine (10T or 30T) with or without 10 μM dCyd (10C) + 100 μM tetrahydrouridine (100THU), a potent competitive inhibitor of cellular cytidine deaminases (23), and compared to nontreated cells (NT). By an allosteric effect on the RNR, the addition of thymidine to the cell culture is expected to modulate the dNTP pool, resulting in an increased [dTTP] and a lower [dCTP] (22, 23, 24).

Early apoptosis (annexin V–positive seven-aminoactinomycin D–negative cells) and late apoptosis/necrosis (annexin-V^+^ seven-aminoactinomycin D–positive cells) were analyzed, showing no thymidine, dCyd, and THU toxicity following treatment of the cells (Fig. S1*A*). To demonstrate that treatment with dThy disrupts the dNTP pool, the expression levels of both RR1 and RR2 subunits of RNR were quantified by quantitative reverse transcription polymerase chain reaction. As shown in Fig. S1*B*, cells treated with 30T exhibited a ∼2-fold increase in *RR1* expression and a ∼1.8-fold increase in *RR2* expression, indicating that cells compensate for nucleotide depletion by upregulating *RNR* expression.

**Figure 4:**
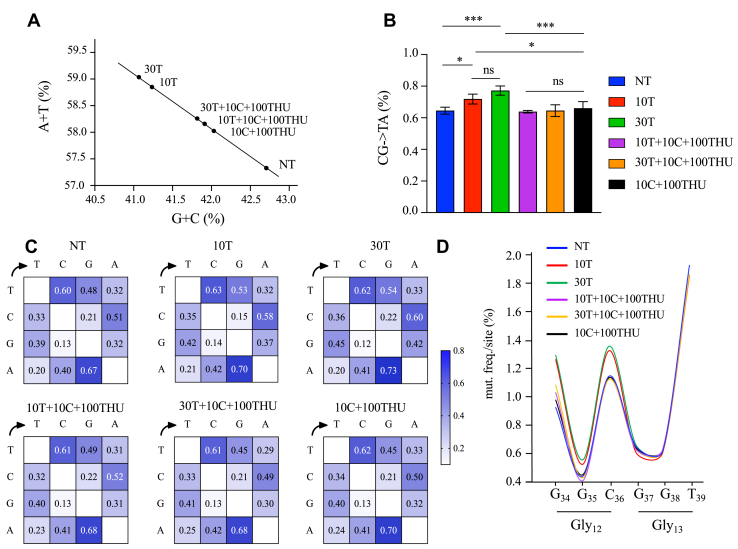
**Analysis of the DNA bases ratios following treatments with thymidine and deoxycytidine.***A*, total DNA was nuclease-digested and deoxynucleosides were analyzed using a Q exactive mass spectrometer. Data were presented as the percentage of total AT and GC nucleotides following treatment of HeLa cell with 10 μM T (10T), 30 μM T (30T), 10 μM T + 10 μM C + 100 μM THU (10T  + 10C  + 100THU), 30 μM T + 10 μM C + 100 μM THU (30T  + 10C  + 100THU), and 10 μM C + 100 μM THU (10C  + 100THU) and were compared to nontreated (NT) cells. *B*, CG- > TA mutation frequencies in HeLa cells treated with 10T, 30T, 10T  + 10C  + 100THU, 30T  + 10C  + 100THU, and 10C  + 100THU and compared to NT cells. Data were subjected to two-way ANOVA, followed by a Sidak *post hoc* test, ∗, *p* < 0.05, ∗∗*p* < 0.01, ∗∗∗, *p* < 0.001, and ∗∗∗∗, *p* < 0.0001, ns: not statistically significant. *C*, heat map representing the mutation matrices of 178 bp of *HRAS* gene following the same treatment described and compared to NT cells, analyzed from NGS data. *D*, site-specific frequency was analyzed for the two hotspots Gly_12_, Gly_13_ present in the exon 2 of *HRAS* and identified by NGS. NGS, next-generation sequencing; NT, nontreated; THU, tetrahydrouridine.

To confirm that dThy treatment increased CG → TA transitions in the human DNA genome, analysis of the DNA bases ratios was performed using a Q exactive mass spectrometer. Data are presented as an average of the percentage of total bases. As shown in Figure 4*A*, a ∼1.03-fold increase, resulting in an enrichment of ∼100 million of A  + T nucleotides within the total DNA, was observed in cells treated with 10T or 30T compared to their respective controls, 10T  + 10C  + 100THU or 30T  + 10C  + 100THU or 10C  + 100THU or NT (Fig. 4*A*).

Increased of CG → TA transitions detected in treated HeLa cells was confirmed by NGS. To evaluate the mutation frequency in NT, 10T, 30T, 10T  + 10C  + 100THU, 30T  + 10C  + 100THU, or 10C  + 100THU, a region of 178 bp surrounding Gly_12_ and Gly_13_ located in exon 2 of HRAS was analyzed by NGS. As observed in Figure 4*B*, the mutation frequency was correlated to the thymidine treatment, with higher mutation frequencies ∼0.72% and ∼0.78% obtained with 10T and 30T, respectively when compared to NT (∼0.65%), cells treated with 10T  + 10C  + 100THU (∼0.63%), 30T  + 10C  + 100THU (∼0.63%), or 10C  + 100THU (∼0.66%) (Fig. 4*B*). Mutation matrices heat maps show the frequency of the different type of mutations following 10T and 30T treatments, with both DNA strands harboring mutations (Fig. 4*C*). Given that *HRAS*^*Gly12*^ and *HRAS*^*Gly13*^ positions are predominantly observed during oncogenesis, the mutation frequencies for these nucleotide positions were analyzed. We noticed that the mutation frequency located at nucleotides G_34_, G_35_, and C_36_ corresponding to Gly_12_, was ∼1.2- to 1.3-fold higher with 10T and 30T (Fig. 4*D*, red and green lines).

To evaluate damages occurring in nuDNA, we analyzed the presence of dsDNA breaks (DSBs), a key feature of tumoral cell transition (31). HeLa cells were incubated with 10T, 30T, 10T  + 10 C  + 100THU, 30T  + 10C  + 100THU, or 10C  + 100THU, and the DSB marker γH2AX was quantified. As observed in Fig. S1*C*, when compared to the positive control (actinomycin), dThy and dCyd treatments did not increased DSB, suggesting that these events were below the technical detection threshold.

Overall, these experiments showed that a [dTTP]/[dCTP] bias induced an excessive DNA alteration with predominant CG → TA mutations. Nonetheless, this process could be bypassed by the addition of dCyd, which counteracts the mutagenic potential of thymidine.

### Deoxynucleotide perturbation impairs mitochondrial metabolism

**Figure 5:**
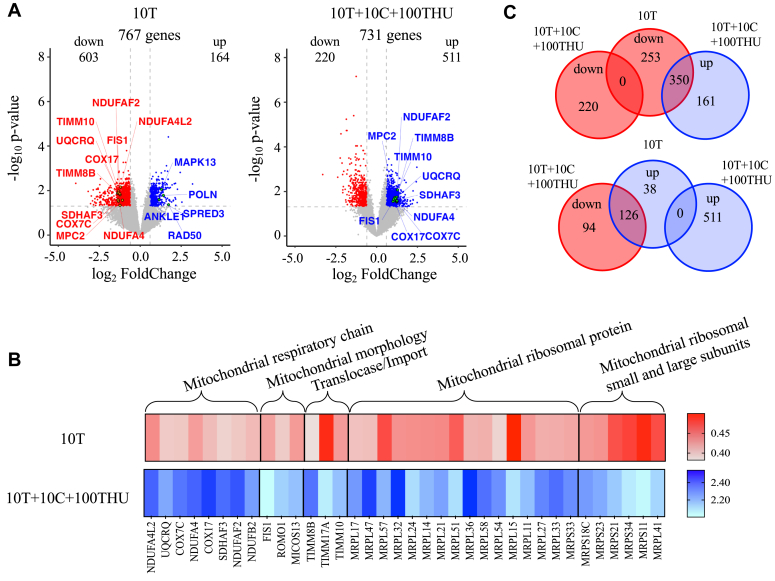
**Distinct nuclear and mitochondrial signatures mediated by thymidine treatment.***A*, volcano plots of differentially expressed genes in HeLa cells treated with 10T or with 10T  + 10C  + 100THU. *Vertical dashed lines* indicate a log2 fold change of 2, and the *horizontal dashed line* indicates a *p* value of 0.001. Data are derived from n = 3 biological replicates. *B*, heat maps of 36 mitochondrial genes induced following *10T* (*red*) or 10T  + 10C  + 100THU (*blue*) treatment in HeLa cells. Groups of upregulated or downregulated genes belonging to the mitochondrial respiratory chain, mitochondrial morphology, mitochondrial ribosomal proteins, and mitochondrial ribosomal small et large subunits. *C*, Venn diagram of significantly upregulated (*blue*) and downregulated (*red*) genes identified with a log2 (fold change) > 2 and a *p* value < 0.001, in HeLa cells treated with 10T or with 10T  + 10C  + 100THU.

To identify thymidine-induced secretion factors involved in cell transformation, gene profiles of HeLa cells incubated with either 10T or 10T  + 10C  + 100THU in comparison to NT cells, were analyzed. To assess the potential functional associations between the genes and biological cellular processes, a NETwork-based Gene Enrichment was performed to highlight the significantly upregulated and downregulated genes. Differentially expressed genes (DEGs) were identified, with a total of 767 and 731 human genes being significantly upregulated and downregulated (adjusted *p* value (Padj) < 0.05 and log_2_ fold change, FC>1 or < -1) for cells treated with 10T and 10T  + 10C  + 100THU, respectively (Fig. 5*A*). Volcano plots generated with the “ggplot” R package present the distribution of all DEGs in cells incubated with 10T or 10T  + 10C  + 100THU (Fig. 5*A*). Interestingly, genes associated with DNA proof-reading and repair mechanism as *MAPK13*, *POLN*, *RAD50*, *ANKLE1*, and *SPRED3* were upregulated in presence of 10T (Fig. 5*A*), suggesting that proof-reading mechanism for mismatches correction were activated.

We noticed also that ∼36 genes involved in the mitochondrial networking, such as the mitochondrial respiratory chain, mitochondrial morphology, import pathways, and mitochondrial ribosomal proteins were downregulated with 10T and upregulated with 10T  + 10C  + 100THU (Fig. 5, *A* and *B*). Although the number of DEG detected between 10T and 10T  + 10C  + 100THU treatments were relatively identical (Fig. 5*A*), the proportion of upregulated and downregulated genes were inversely related between the two conditions (Fig. 5*C*). This suggests that the treatment triggered opposite effects and highlights a potential protective effect of the dCyd. Taken together, these data showed that thymidine treatment induced a spectrum of perturbation affecting the mitochondrial network, a phenomenon which could be counteracted in presence of dCyd.

**Figure 6:**
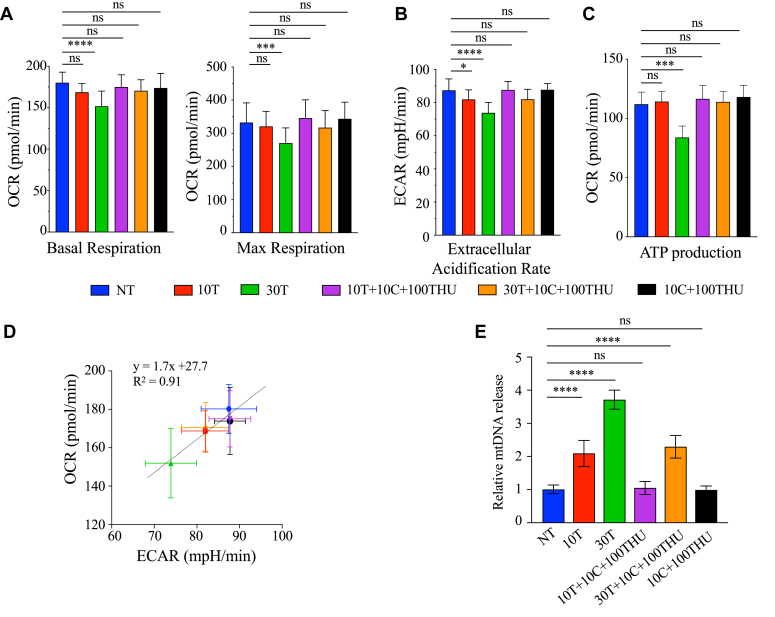
**Thymidine disrupted mitochondrial network and cellular metabolism.***A*, measure of the oxygen consumption rate (OCR) in HeLa cells treated with 10T, 30T, 10T  + 10C  + 100THU, 30T  + 10C  + 100THU, and 10C  + 100THU and compared to nontreated (NT) cells. The data obtained in Fig. S2 were used to calculate the basal and max respiration. *B*, the extracellular acidification rate (ECAR) was analyzed using the data obtained in Fig. S2. *C*, Metabolic phenotype indicative of the bioenergetics state of mock- and HeLa-treated cells after 18 h, generated through OCR and extracellular acidification rate (ECAR) values under basal (normal) conditions and treated conditions. *D*, HeLa-treated cells were fractioned and mtDNA quantification was performed using the *MT-COI* gene in the cytosolic fraction and normalized to the quantification of the nuclear gene *β2M* of the total fraction. Data obtained in A, B, and E were subjected to two-way ANOVA, followed by a Sidak *post hoc* test, ∗, *p* < 0.05, ∗∗*p* < 0.01, ∗∗∗, *p* < 0.001, and ∗∗∗∗, *p* < 0.0001, ns: not statistically significant. MT-COI, mitochondrial cytochrome c oxidase subunit I; mtDNA, mitochondrial DNA.

To assess the effect of thymidine on cellular bioenergetics, HeLa cells were incubated with 10T, 30T, 10T  + 10C  + 100THU, 30T  + 10C  + 100THU, 10C  + 100THU, and NT cells were used as a negative control. At 16 h post treatment, an analysis of mitochondrial extracellular fluxes was performed using the Seahorse extracellular flux analyzer (Fig. S2*A*). The oxygen consumption rate (OCR), the production of ATP and the extracellular acidification rate (ECAR) were evaluated, as they are representative indicators of mitochondrial oxidative phosphorylation system (OXPHOS) and lactic acid production. As observed in Figure 6*A* and Fig. S2*B*, 30T cells treatment induced a decrease in basal and maximal mitochondrial respiration. Indeed, a ∼1.22 and ∼1.18-fold decrease of maximal respiration and spare respiratory capacity, respectively, was observed with 30T treatment but remained basal state when cells were treated with both 10T  + 10C  + 100THU or 30T  + 10C  + 100THU (Figs. 6*A*, S2*C*). Lactic acid measurement (ECAR) was also decreased with 10T and 30T (Figs. 6*B* and S2*D*). We notice that 30T leads to a marked drop ∼1.4-fold decrease in ATP levels, which could indicate that the cells are under stress (Fig. 6*C*). The induced shift of the metabolic phenotype in 10T- and 30T-treated cells, toward a lower energetic state was further visualized in a metabolic phenogram by plotting the OCR against the ECAR values under basal and treated conditions (Fig. 6*D*). Treatment with 10T and 30T decreased the OCR/ECAR ratio in a dose-dependent manner, as compared to NT cells or cells treated with 10T  + 10C  + 100THU or with 30T  + 10C  + 100THU, pointing the decreased in mitochondrial metabolic activity (Fig. 6*D*). Interestingly, as previously observed, mitochondrial bioenergetics remained at the basal state in the presence of 10T  + 10C  + 100THU or 30T  + 10C  + 100THU.

We next examined the implication of thymidine and dCyd on the damage of mitochondrial network as the disruption of energy production, alteration of mitochondrial dynamics, and the release of mtDNA. For this, HeLa cells were treated with 10T, 30T, 10T  + 10C  + 100THU, 30T  + 10C  + 100THU, or 10C  + 100THU and released mtDNA was quantified. At 72 h posttreatment, HeLa cells were subjected to digitonin fractionation, and whole-cell extracts (WCEs) and cytosolic extracts were blotted using specific antibodies. As visualized in Fig. S3*A*, when compared to β-actin and MAP/ERK kinase-1/2 (MEK1/2) two cytosolic proteins, apoptosis-inducing factor (AIF) and Histone H3, specific markers for mitochondria and nuclei, respectively, were absent in the cytosol fraction, demonstrating that the purification of the cytosol was not contaminated by remaining mitochondrial or nuclear proteins.

Quantification of mtDNA was performed by qPCR using mitochondrial cytochrome c oxidase subunit I (*MT-COI*) gene in the cytosolic fraction and further normalization using nuclear β2M gene in the WCE fraction. MtDNA in cytosolic extracts was increased in a dose-dependent manner by up to ∼2- and ∼3.7-fold (Fig. 6*E*) following treatment with 10T and 30T, respectively, indicating a release of mtDNA. This phenomenon is counteracted by adding 10T  + 10C  + 100THU and lower with 30T  + 10C  + 100THU. As a control, mtDNA levels in WCE were quantified, revealing consistent levels across all tested samples. This indicates that the various treatments do not reduce cellular mtDNA content but rather influence its distribution within the cells (Fig. S3*B*). These results highlight that the increased mtDNA release points to damage mitochondria following thymidine treatment, potentially leading to mitochondrial dysfunction.

To investigate whether mtDNA could also undergo mutations due to disruptions in the intracellular nucleotide pool, HeLa cells were treated with 10T, 30T, 10T  + 10C, 30T  + 10C, and 10C  + 100THU for 24 h. Total DNA was extracted from each sample and analyzed by differential DNA denaturation PCR (3DPCR), a selective PCR amplification designed to detect AT-rich fragments (32), based on the fact that AT-rich sequences denature at lower temperatures than GC-rich sequences during PCR. A nuDNA segment (*TP53*) and a mtDNA segment (*MT-COI*) were analyzed using denaturation temperature (Td) gradients of 92 °C to 86 °C for *TP53* and 88 °C to 78 °C for *MT-COI*. As illustrated in Fig. S3*C* among all conditions tested, only the 30T-treated samples exhibited significantly lower Td values, 88 °C for *TP53* and 83.3 °C for *MT-COI*. Mutation matrices analyzed at Td values of 88.8 °C for *TP53* and 84.7 °C for *MT-COI* revealed an enrichment in A  + T mutations specifically in the 30T-treated samples consistent with their lower Td values. These finding demonstrate the mutagenic effect of dThy (Fig. S3*D*).

Overall, these data indicate that unbalanced dNTP pools can affect the nuDNA, mtDNA, and perturb the mitochondrial network. Both nuDNA and mtDNA are susceptible to damage, with mitochondria possessing limited DNA repair capabilities. Fluctuations in dNTP levels affect the ability to repair mutated nuDNA and mtDNA, which in concert with the release of mtDNA, would lead to dysfunctions in mitochondrial metabolism. However, this phenomenon could be counteracted with equilibrated concentration of dNTPs.

### Detection of mutated *KRAS* genes based on the loss of hydrogen bonds

As *KRAS* gene mutations located at positions Gly_12_ and Gly_13_ are particularly associated to the progression of oncogenesis, we screened the occurrence of these two mutations in tumors and healthy tissues of 135 patients with a colorectal cancer. For these patients, we reported the location of the tumor in the colon, the stage of the tumor, the tumor-node-metastasis staging, the presence of vascular invasion, the occurrence of infiltrations of T cells expressing naive/memory markers CD3^+^, CD8^+^ CD45RO^+^, and the percentage of tumoral cells (Table S1). Pyrosequencing identified the presence of somatic alterations in *KRAS* (39/135, ∼29%), *BRAF* (19/135, ∼14%), or *PI3K* (16/135, ∼12%) genes and the bisulfite treatment determined the *MLH1*, *HERV*, and *LINE-1* methylation patterns (Table S1).

Since *KRAS* mutations are detected in the sequence coding for Gly_12_ and Gly_13_ and frequently associated with CG → TA transitions, we performed a screening based on the melting temperature (Tm) variation during SYBR Green amplification. Here, the occurrence of a transition leads to the loss of hydrogen bonds. As the Tm variation is correlated to the number of hydrogen bonds, the occurrence of a single CG → TA point mutation could selectively differentiate between mutated and non-mutated *KRAS*. This selective amplification was visualized by SYBR Green across a short amplification window of 30 bp that selects the different *KRAS* alleles as a function of the Tm.

**Figure 7:**
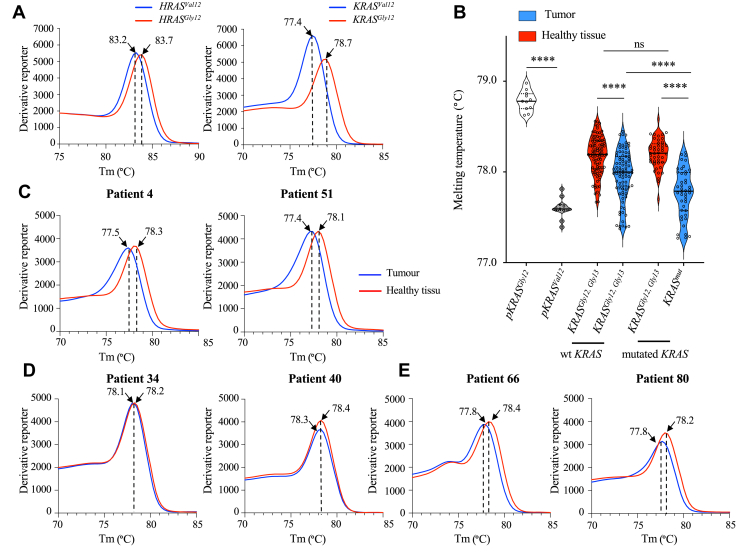
**Selection of *KRAS* mutated gene in patients by SYBR Green.***A*, SYBR *Green* melting profiles was performed on a *KRAS* 30-nt window located in exon two and covering the two hotspots, Gly12 and Gly13 sites. Amplification was realized for *HRAS*^*Gly12*^, *HRAS*^*Val12*^, *KRAS*^*Gly12*^, and *KRAS*^*Val12*^. *B*, amplification by SYBR *Green* was carried out on 135 patients with colorectal cancer. Samples were separated according to tumor and healthy tissues. A positive and negative control was performed with *KRAS*^*Gly12*^ and *KRAS*^*Val12*^ DNA, respectively. Data were subjected to two-way ANOVA, followed by a Sidak *post hoc* test, ∗, *p* < 0.05, ∗∗*p* < 0.01, ∗∗∗, *p* < 0.001, and ∗∗∗∗, *p* < 0.0001, ns: not statistically significant. *C*–*E*, Tm is indicated on the *x*-axis and fluorescence quantification is reported on the *y*-axis. SYBR *Green* melting profiles were performed on patients with colorectal cancer and compared to their own healthy tissue, (*C*) patient with a *KRAS*^*Val12*^ mutation, (*D*) patient with no mutations, (*E*) patient with a mutation located in different sites of Gly12 and Gly13.

Optimizations were first carried out on the wild-type *H/KRAS*^*Gly12*^ and mutated *H/KRAS*^*Val12*^ to validate the SYBR Green amplification efficiency. As observed in Figure 7*A*, Tm of 83.2 °C and 83.7 °C were obtained for *HRAS*^*Val12*^ and *HRAS*^*Gly12*^ genes, respectively. For *KRAS*^*Val12*^ and *KRAS*^*Gly12*^ alleles, the Tm was in the range of 77.4 °C and 78.7 °C, respectively. The variation of ∼5 °C in the melting profiles between *HRAS* and *KRAS* is related to the different in GC base composition between *HRAS* (∼59.1%) and *KRAS* genes (∼37.6%) (Fig. S4).

Optimized SYBR Green condition were then performed on the cohort of patients, subdividing them into four groups based on healthy and tumor tissues possessing WT *KRAS (KRAS*^*Gly12, Gly13*^*)* or mutated *KRAS* gene (*KRAS*^*mut*^), corresponding to the different types of substitutions in Gly_12_ or Gly_13_. As visualized in Figure 7*B*, we observed a significant decrease in the Tm for the patients mutated in the *KRAS* cohort, as compared to their healthy tissues. Interestingly, a slightly reduced Tm was detected in the group of patients having a nonmutated *KRAS* at the tumor level compared to their corresponding healthy tissues, suggesting that other CG → AT mutations located at different nucleotide positions of Gly_12_ and Gly_13_ could be present.

To validate these data, we analyzed in detail 2 patients in each group, having either a mutation in the *KRAS* gene (patients 4 and 51, Fig. 7*C*), or a nonmutated *KRAS* gene (patients 34 and 40, Fig. 7*D*), or a nonmutated *KRAS* gene in position Gly_12_ or Gly_13_ differing in the Tm (patients 66 and 80, Fig. 7*E*). These data clearly showed that the occurrence of mutations located at position Gly_12_, or Gly_13_ or at any other position could influence the melting profile. To further confirm the specificity of the essay, the presence of mutations located in *KRAS* gene has been validated by direct sequencing in tumors (Fig. S5*A*) and in healthy tissues (Fig. S5*B*). Overall, these data indicate that the loss of hydrogen bonds in any position, related to CG- > TA mutations, allows Tm-dependent detection of mutated *KRAS* genes.

## Discussion

**Figure 8:**
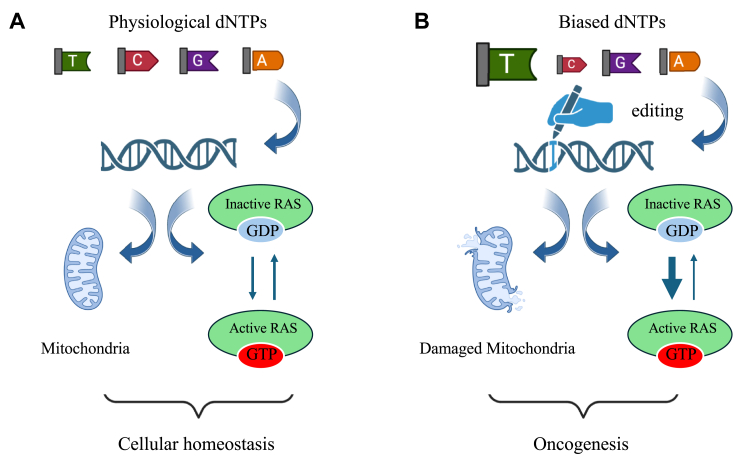
**Perturbation of dNTPs pools leads to oncogenesis and disruption of mitochondrial metabolism.***A*, physiological situation with a balanced dNTP pool. *B*, fluctuation on intracellular dNTP pool alters the nuclear DNA and mitochondrial metabolism.

The present data indicate that unbalanced dNTPs concentration impede DNA replication to proceed with the highest fidelity. Fluctuation of dNTPs increases the mutation frequency across the whole genome, including *RAS* gene at codons for glycine 12 and 13, known hotspots in cancers and leads to disruption of mitochondrial metabolism (Fig. 8). Multiple data sources provide compelling evidence that variations within somatic cell pools significantly impact spontaneous mutation rates and increase sensitivity to DNA-damaging agents (33).

The accumulation and the functional consequences of replication errors are one of the main endogenous factors influencing genomic instability. In living systems, a high burden of random mutagenesis could result in the emergence of cancers. This multistep outcome has several genetic or environmental origins, among which combine two distinct biological mechanisms, misincorporation of a noncomplementary base during replication, and the subsequent failure of proof-reading systems to correct these errors.

Analysis of the PCR mutagenized *HRAS* gene demonstrated that an increased [dTTP] and decreased [dCTP] concentrations generated a swarm of mutants with significant transition frequencies of CG → TA occurring in a dose-dependent manner (Fig. 1, *B* and *C*). Functionally, a 100/1 bias for [dTTP] and [dCTP] allowed selection of *HRAS*^*Cys12*^ mutant harboring a phenotype associated to alteration of cell morphologies (Fig. 2*A*). Conservation of Gly_1*2*_ appears to be fundamental in preserving the cellular physiological function of *HRAS*, and the replacement of this amino acid by any other, except Pro, are transforming. An observation highlighting the prevalent role of this position during the oncogenesis process (34).

The protein structural analysis (Fig. 3) of the different *HRAS* mutants suggested that the mutations located in position HRAS^Ala11Val^, HRAS^Gly12Cys^, HRAS^Thr74Ile^, HRAS^Cys186Tyr^, and HRAS^Val187Met^ were structurally neutral (Fig. 3, *B* and *C*). Furthermore, functional assays demonstrated that apart from HRAS^Gly12Cys^, no other mutation of HRAS alters the catalytic activity of the enzyme (Figs. 2 and 3).

Thymidine supplemented to the culture medium may be scavenged by the cell and phosphorylated to yield the triphosphate. This not only increases intracellular [dTTP] but, by its allosteric effect on the RNR enzyme, specifically decreases CDP reduction to dCDP, leading to a decline in intracellular [dCTP] (22, 23, 24). Interestingly, treatment of HeLa cells with 30T induced an upregulation in the expression of *RR1* and *RR2* subunits (Fig. S1*B*). Increased dThy concentration, causing dNTP imbalance, likely exerts pressure on the cell to elevate *RR1* and *RR2* RNR subunit expression in an attempt to restore this balance. Thus, the observed increase in RR1 and RR2 levels may reflect a cellular response aimed at maintaining the dNTP levels necessary for DNA replication and repair. HeLa cells incubated with dThy showed an increased mutation frequency in *HRAS* gene (Fig. 4, *B* and *C*). Other mutations throughout the whole genome were enriched by ∼100 millions in A  + T nucleotides within the genome (∼1.029-fold increase, Fig. 4*A*). Given that the number of mutations calculated for all types of cancer can vary between ∼20 to >1 million (35), we can therefore suggest that the fluctuation of the dNTP pool will generate a significantly higher number of mutations.

Many factors could potentially contribute to mitochondrial metabolism failure. In particular, cellular dNTP pool fluctuations could lead to the genomic instability of nuDNA and mtDNA (31). Following nucleotide imbalances, transcriptomic analysis (Fig. 5, *A* and *B*), showed a number of perturbations occurring within the mitochondria. Targeted genes were involved in mitochondrial respiratory chain, the mitochondrial morphology, the mitochondrial ribosomal proteins, or the mitochondrial ribosomal small and large subunits being altered (Fig. 5*C*). Interestingly, it has been described that the maintenance of mitochondrial genome is under the control of nuDNA, and when damaged, can cause depletion or multiple alterations of mtDNA (36, 37, 38, 39). Throughout evolution, the mitochondria have lost their genetic autonomy and the mtDNA has become dependent on numerous factors encoded by the nucleus for its integrity, replication, and gene expression. Mutations in any of these factors could disrupt communication between the two genomes and potentially promote diseases by affecting the integrity or expression of mtDNA (40).

Among mitochondrial metabolism, we assessed the effect of dThy on cellular bioenergetics by analyzing extracellular fluxes using the Agilent Seahorse technology to evaluate the ECAR and the OCR, which are representative indicators of lactic acid production and mitochondrial OXPHOS, respectively. Figure 6 confirmed the role of fluctuation in dNTPs leading to a lower maximum capacity in mitochondrial functions. A simultaneous drop in OXPHOS, ATP, and acid lactic productions suggests that cells are unable to compensate, and could indicate in response to dNTP imbalance, a severe cellular damage or a state of major stress. Along with these observations, we noticed that the decreased mitochondrial function led to the release of mtDNA. These effects induced by dThy treatment could be counteracted by dCyd (Fig. 6*E*).

To confirm the clinical relevance of these observations, we explored colorectal cancers where the mutation burden is well established (41). We showed that *KRAS* genes differing by a single or several CG → TA substitutions can be selectively amplified (Fig. 7). The PCR, based on SYBR Green fluorescence with no specific allele oligonucleotides need, is well adapted for SNP detection. As *KRAS* mutations are an early event in transformation, detection at the Gly_12_ position could be an effective tool to diagnose the presence of malignant cells at the early stage (42). As the window of observation is ∼30 bases, amplification could detect any mutation associated with a decrease in hydrogen bonds and be correlated to a reduced Tm. As CG → TA substitutions represent the most frequent substitutions (40–50%) characterizing human cancers (35), it would be interesting to evaluate this method to detect specific mutations characteristic of a circulating tumor cells in a blood sample (43).

Among the detected mutations in tumors analyzed in >7000 cancers, more than half of the mutations are CG → TA transitions, suggesting that a bias in the [dTTP] and [dCTP] could be a key player in the appearance of mutations (35). It is now well established that in cancers such as colorectal, gastric, and endometrial cancers, display specific signatures (44). In these settings, defective DNA mismatch repair and dNTP unbalance could be hardly discernible (35). Different signatures associated with proo-freading deficiency have been referenced highlighting a high frequency of CG → TA mutations, confirming that fluctuation of dNTP pool and defective system repair could be a potent source of mutations. Our observations further show that unbalances of dNTP pools might contribute to mutagenesis of nuclear and mtDNA, triggering altered mitochondrial network function, a contributor to oncogenesis.

Fluctuations in the dNTP pool are a critical factor in genetic instability, significantly contributing to the emergence of mutations and potentially driving cancer development. Altered dNTP levels compromise genome integrity, increasing replication errors, and enhancing sensitivity to DNA-damaging agents. A deeper understanding of these mechanisms could pave the way for innovative therapeutic strategies targeting dNTP regulation, with the goal of limiting the accumulation of oncogenic mutations and, ultimately, preventing tumor progression.

## Experimental procedures

### Patients

The human studies reported on in the present work abide by the Declaration of Helsinki principles. This study was approved by the ethics committees of the “Centre Hospitalier Régional Universitaire” of Tours. Tumor samples came from a cohort of colorectal cancers collected in the pathology department of the University Hospital of Tours. The collection contains frozen and formalin-fixed, paraffin-embedded tumor material, as well as paired normal tissue; written informed consent was obtained from all patients. According to French laws and recommendations, the collection has been declared to the French Ministry of Scientific Research and is registered under N° DC-2008-308. We constituted an exploratory cohort of 135 selected patients who had undergone surgery for a colorectal cancer between January 2007 and May 2008 at the Trousseau hospital of Chambray-lès-Tours. Obtaining individual written consent prior to use of each sample, as recommended by the institutional guidelines, was required.

### Reagents

Thymidine (#89270), 2′-dCyd (#3897), THU (#584223), mouse monoclonal anti-β-actin-peroxidase antibody (#A3854), and individual dNTPs for routine PCR (DNTP100A) were purchased from Sigma-Aldrich. BioTaq DNA polymerase was from Meridian Bioscience (BIO-21040). Anti-MEK1/2 (D1A5) rabbit antibody (#8727), anti-AIF (D39D2) XP rabbit antibody (#5318), anti-Histone H3 (D1H2) XP rabbit antibody (#4499), anti-P-Histone H2AX (S139) (20E3) rabbit antibody (Alexa 488) (#9719), and anti-rabbit IgG horseradish peroxidase-linked antibody (#7074) were from Cell Signaling Technology. eBioscience Annexin V Apoptosis Detection Kit-eFluor 450 (88-8006-74) was from Invitrogen. NucleoSpin Gel and PCR Clean-up kit (#740609) was from Macherey-Nagel. Quant-iT dsDNA Assay kit, High sensibility (#Q33120) was from Invitrogen, NEBNext DNA Library Prep kit (#E7645), and NEBNext Multiplex Oligos for Illumina (#E7600) were from New England BioLabs. TOPO TA cloning Kit (pCR 2.1- TOPO Vector, #45-0641) was from Invitrogen.

### Cell culture

Human HeLa (CRM-CCL2) and NIH/3T3 (CRL-1658, mouse embryonic fibroblasts) cells were maintained in Dulbecco’s modified Eagle’s medium (Gibco), supplemented with heat-inactivated fetal calf serum (10%), penicillin (50 U/ml), and streptomycin (50 mg/ml), and were grown in 75-cm^2^ cell culture flasks in a humidified atmosphere containing 5% CO_2_.

### PCR, 3DPCR, and PCR mutagenesis

The first reaction of *HRAS* cDNA involved standard amplification, the reaction parameters were 95 °C for 5 min, followed by 35 cycles (95 °C for 30 s, 55 °C for 30 s, and 72 °C for 5 min) and finally for 10 min at 72 °C. The primers used to amplify the *HRAS* cDNA were *HRAS*ext fwd and *HRAS*ext rev. PCR products were purified from agarose gels (NucleoSpin Gel and PCR Clean-up kit, Macherey-Nagel) and ligated into the TOPO TA cloning vector (Invitrogen). After transformation of Top10 electrocompetent cells (Invitrogen), up to 15 clones were picked. Sequencing was outsourced to Eurofins to validate that the *HRAS* cDNA was not mutated. From this *HRAS* construct, mut-PCR reactions were carried out in the following reaction mixture, 1 × NH4 reaction buffer, 2.5 mM MgCl_2_, 100 μM of each primer, and 5U Taq polymerase (meridian Bioscience). Different dNTP concentrations were used. In all PCR conditions [dTTP] was used at 1 mM, [dGTP] and [dATP] were at 50 μM and [dCTP] used at 1000, 100, 50, 30, and 10 μM. Primers used were designed *HRASHindIII* fwd and *HRASKpnI* rev. The cycling parameters were: 50x (95 °C, 30 s; 60 °C 30 s; 72 °C 10 min). Long elongation times were used to favor elongation after mismatches. PCR-amplified material was purified on agarose gels, cleaved by *HindIII* and *KpnI*, and ligated with similarly digested and dephosphorylated expression plasmid pSV2-gpt for the NIH-3T3 electroporation. *HRAS1*^*Ala11Val*^ and *HRAS1*^*Gly12Cys*^ were generated by site-directed mutagenesis (GeneArt Site-Directed Mutagenesis System, Thermo Fisher Scientific).

Differential DNA 3DPCR was performed on an Eppendorf gradient Master S programmed to generate an 86 to 92 °C or 78 to 88 °C gradient in the Td for *TP53* and *MT-COI* regions, respectively. This technique relies on the fact that AT-rich DNA denatures at a lower temperature than GC-rich DNA. A fragment of *TP53* (176 bp) and *MT-COI* (245 bp) were amplified by employing a nested procedure. First PCR conditions were: 5 min 95 °C then (1 min 95 °C, 1 min 58 °C, 2 min 72 °C) × 42 cycles. PCR products were purified from agarose gels (NucleoSpin Gel and PCR Clean-up, Macherey-Nagel). Nested PCR was performed with 1/100 of the purified first round PCR products, amplification conditions were: 5 min 95 °C then (30 s, 86–92 °C or 78–88 °C, 30 s 58 °C, 1 min 72 °C) × 45 cycles, and then 20 min 72 °C. 3DPCR products were purified from agarose gels (NucleoSpin Gel and PCR Clean-up, Macherey-Nagel) and ligated into the TOPO TA cloning vector (Invitrogen), and ∼40 to 45 colonies were sequenced. All the primers are presented in Table S2.

### Quantitative PCR

Total DNA was extracted using MasterPure Complete DNA and RNA extraction kit (Biosearch Technologies) according to the manufacturer’s instructions. Real-time quantitative PCR based on SYBR Green (Power SYBR Green PCR Master Mix, Applied Biosystems) was performed using DNA samples. Conditions were 2 min at 50 °C then 10 min at 95 °C then 20 s at 95 °C, 20 s at 55 °C, and 1 min at 68 °C for 40 cycles. Primers used for *HRAS* amplification were *HRAS*SYBR fwd and *HRAS*SYBR rev, for *KRAS* amplification *KRAS*SYBR fwd and *KRAS*SYBR rev, for mtDNA (*MT*-*COI*) amplification *dHCoxI* fwd and *dHCoxI* rev and for nuDNA (*β2M*) amplification *β2M* fwd and *β2M* rev. Primers are described in Table S2.

### Subcellular fractionation

5.10^6^ cells were collected 24 h posttreatment, washed in PBS and split: 10^6^ cells were kept for Western Blot on WCE, 10^6^ cells were kept for total DNA extraction, and 3.10^6^ cells were lysed in 1 ml digitonin buffer (100 μg/ml digitonin, 150 mM NaCl, 50 mM Hepes (pH 7), protease and phosphatase inhibitors cocktail) for 15 min at 4 °C. After centrifugation (2000*g*, 10 min, 4 °C) the supernatant was centrifuged three times (20000*g*, 20 min, 4 °C), the last supernatant constitutes the cytosolic fraction and was split in two, one half for DNA extraction, the other for Western blotting. The initial pellet was resuspended in 300 μl NP-40 buffer (1% NP-40, 150 mM NaCl, 50 mM Hepes(pH 7), protease and phosphatase inhibitors cocktail), for 30 min at 4 °C. After centrifugation (7000*g*, 10 min, 4 °C) the pellet and supernatant were split in half for Western blotting and DNA extraction, the pellet constitutes the nuclear fraction and the supernatant constitutes crude mitochondrial lysate.

### Electroporation

NIH/3T3 cells were transiently transfected by electroporation. Briefly, NIH/3T3 cells were harvested by trypsin-EDTA, resuspended at a density of 10^6^ cells in 0.2 ml in an electroporation cuvette (Invitrogen) and transfected by applying a 330 V pulse using a pulse generator (Electroporator II; Invitrogen). NIH-3T3 were electroporated with pSV2-*HRAS* carried out with 10% fetal calf serum and antibiotics. All electroporations were done in triplicate. A library of pSV2-Ha-Ras performed with biased dNTP was electroporated. Controls were accomplished with 15 μg of *HRAS*^*Gly12*^ and *HRAS*^*Val12*^ as negative and positive controls, respectively. Electroportation efficiency, ∼80% was determined by fluorescent-activated cell sorting analysis following electroportation of 15 μg of *GFP* plasmid. Culture was started the first day with 10% fetal calf serum and at day 3 postelectroporation, cells were washed with PBS, trypsinized and divided in two flasks and complemented with 2% fetal calf serum. At 7 days post electroporation cells were grown without fetal calf serum. Three weeks post electroporation transformed cells appeared *HRAS*^*Val12*^ and with the *HRAS*^*Gly12*^ mutagenized electroporated library. DNA from transformed cells was extracted, PCR amplified and cloned in a TOPO TA vector to confirm the obtained mutations. New constructions were performed from the detected *HRAS* sequences and cloned in the pSV2-gpt plasmid *via HindIII* and *KpnI* sites and individually electroporated in NIH-3T3 murine cell line. New transformed cells appeared 3 weeks post electroporation.

### Western blotting

Cells were treated with 1X radio-immunoprecipitation assay lysis buffer (#20-188, Merck Millipore) and Complete ETDA-free Protease Inhibitor (#11873580001, Merck Millipore), followed by sonication and centrifugation at 20,000*g* for 10 min. Clarified lysates were loaded on NuPAGE 4 to 12% Bis-Tris Gel (Invitrogen) and transferred to a nitrocellulose membrane (Invitrogen) according to the manufacturer’s instructions. Primary antibodies (1/1000) were incubated overnight in 2.5% bovine serum albumin, followed by horseradish peroxidase-linked secondary antibodies (1/5,000) incubation for 2 h. Membranes were revealed using a chemiluminescence assay (Thermo Fisher Scientific). Mouse monoclonal anti-B-actin-peroxidase antibody (1/50,000) was used as a loading control. Purity of cellular fractionation was confirmed using anti-MEK1/2 as a cytosolic protein marker, anti-AIF as a mitochondrial marker, and anti-Ηistone H3 as a nuclear protein marker (1/1000). The cellular fractionation validation was performed on whole cell lysates as well as cytosolic partitions.

### Digestion and HPLC analysis of product

DNA was then digested to single nucleotides using a Nucleoside Digestion Mix (New England BioLabs). Analysis of DNA bases ratios were performed using a Q exactive mass spectrometer (Thermo Fisher Scientific). The instrument was equipped with an electrospray ionization source (H-ESI II Probe) coupled to an Ultimate 3000 RS HPLC (Thermo Fisher Scientific). A ThermoFisher Hypersil Gold aQ chromatography column (100 mm ∗ 2.1 mm, 1.9 μm particle size) heated to 30 °C was injected with digested DNA. The flow rate was set to 0.3 ml/min and the column was run for 10 min in isocratic eluent consisting of 1% acetonitrile in water containing 0.1% formic acid. Parent ions were fragmented in positive ion mode, parallel reaction monitoring mode at 10% normalized collision energy; tandem mass spectrometry resolution was 17,500, automatic gain control target was 2e5, maximum injection time was 50 ms, and separation window was 1.0 *m/z*. The inclusion list contained the following masses: dC (228.1), 5-mdC (242.1), dA (252.1), dG (268.1), and dT (243.1). Extracted ion chromatograms (±5 ppm) of basic fragments were used for detection and quantification (dC: 112.0506, 5-mdC: 126.0662, dA: 136.0616, dG: 152.06565, dT: 127.0501). Calibration curves were previously generated using synthetic standards in the range of 0.2 to 50 pmol injected. Results were expressed as the percentage of total bases.

### Deep sequencing

For the analysis of mutations in *HRAS* gene using biased dNTPs or cells treated with the different drugs, amplification was performed by standard PCR using the primers *SEQRAS*mut fwd and *SEQRAS*mut rev or *SEQRAS*deep fwd and *SEQRAS*deep rev (Table S1). Conditions were 5 min at 95 °C, then 35 cycles of 30 s at 95 °C, 30 s at 60 °C, and 1 min at 72 °C, followed by 10 min at 72 °C. PCR products were purified with the NucleoSpin Gel and PCR Clean-up kit (Macherey-Nagel) and quantified using the Quant-iT dsDNA Assay Kit, High sensibility (Invitrogen) according to the manufacturer’s instructions. The preparation of the DNA library was performed with the NEBNext DNA Library Prep Kit and NEBNext Multiplex Oligos for Illumina (New England BioLabs) for the fragmentation and multiplex adapters’ ligation steps. Deep sequencing was performed with Illumina cBot and GAIIX technology.

Sequenced reads were cleaned of library adapter and base pairs occurring at 5′ and 3′ ends with a Phred quality score <20 were trimmed off by AlienTrimmer (45) (https://gitlab.pasteur.fr/GIPhy/AlienTrimmer). Reads with a minimum length of 35 were subject for the next analysis. For the SNP analysis, duplicated reads were removed with Picard-tools v2.23.3 (https://broadinstitute.github.io/picard/; Broad Institute). Single nucleotide variants (SNP) detection was performed with bcftools mpileup V1.13 (46) to extract and detect their occurrence. This workflow of analysis was resumed in a nextflow workflow (47).

### RNA sequencing

The RNA-seq analysis was performed with Sequana. In particular, we used the RNA-seq pipeline (v0.15.2, https://github.com/sequana/sequana_rnaseq) built on top of Snakemake v7.25.0 (48). Reads were trimmed from adapters and low-quality bases using fastp software v0.22.0 (https://gitlab.pasteur.fr/GIPhy/AlienTrimmer) (49), and then mapped to the human genome using STAR v2.7.10b (50). Genomes and annotations were downloaded from Ensembl website using hg38 reference. FeatureCounts 2.0.1 (51) was used to produce the count matrix, assigning reads to features using annotation aforementioned. Statistical analysis on the count matrix was performed to identify differentially regulated genes. Differential expression testing was conducted using DESeq2 library 1.34.0 (http://www.bioconductor.org/packages/release/bioc/html/DESeq2.html) (52) scripts, and HTML reporting made with the Sequana RNA-seq pipeline. Parameters of the statistical analysis included the significance (Benjamini–Hochberg adjusted *p* values, false discovery rate < 0.05) and the effect size (fold change) for each comparison.

## Data availability

The data discussed in this work have been deposited in NCBI’s Gene Expression Omnibus and are accessible through GEO Series accession number GSE261897.

## Supporting information

This article contains supporting information.

## Conflict of interest

The authors declare they have no conflicts of interest with the contents of this article.
