# Supplementary material for: Disruption of deoxyribonucleotide triphosphate biosynthesis leads to RAS proto-oncogene activation and perturbation of mitochondrial metabolism
Source: J Biol Chem. 2024 Dec 23;301(2):108117. doi: 10.1016/j.jbc.2024.108117 (PMC11791277; doi:10.1016/j.jbc.2024.108117)
Supplement: Supporting Figure S1 [file mmc1.pdf]

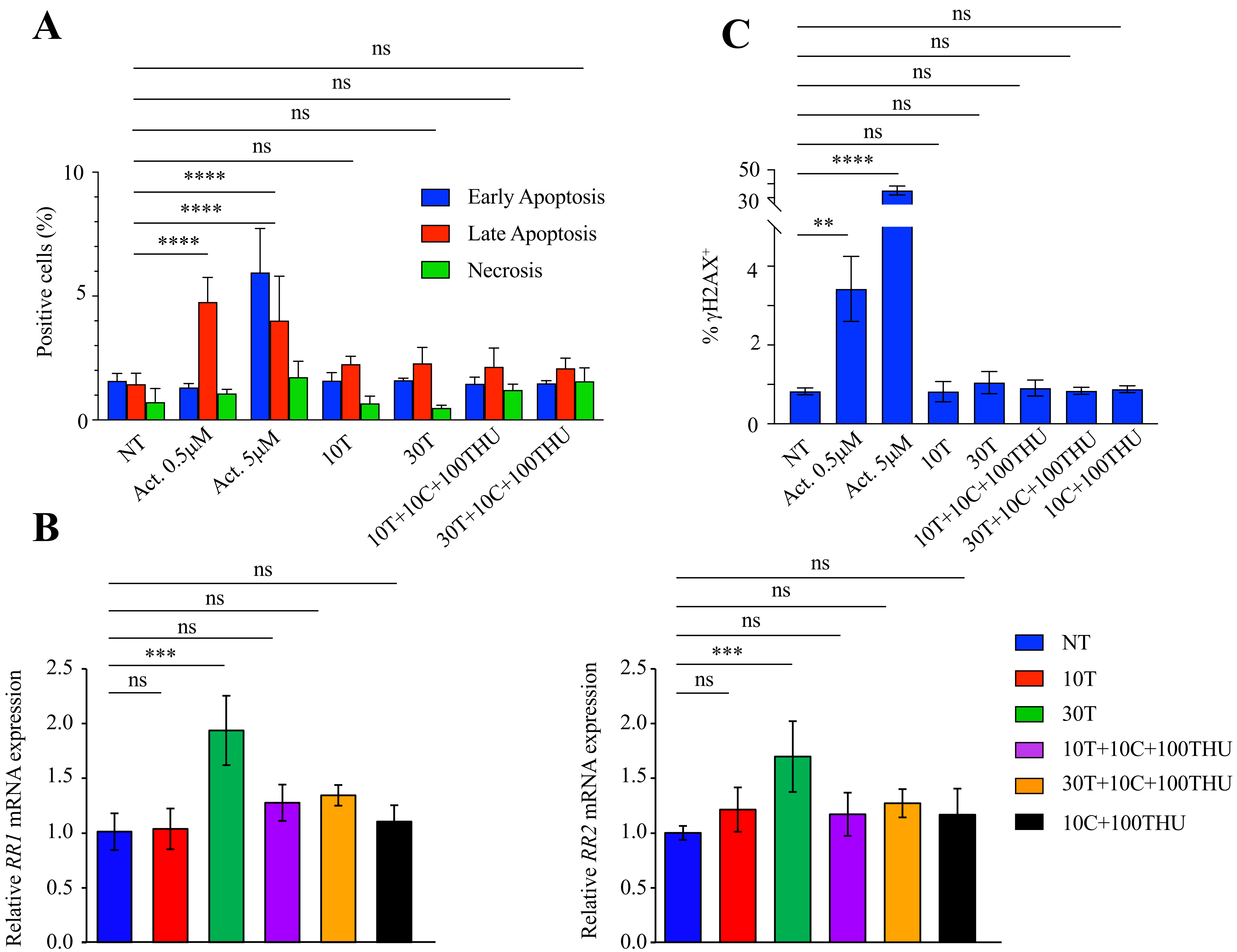

A
ns
ns
ns
ns
****
****
****
10
5
0
Positive cells (%)
Early Apoptosis
Late Apoptosis
Necrosis
NT
Act. 0.5μM
Act. 5μM
10T
30T
10T+10C+100THU
30T+10C+100THU
C
ns
ns
ns
ns
ns
****
**
50
30
4
2
0
% γH2AX+
NT
Act. 0.5μM
Act. 5μM
10T
30T
10T+10C+100THU
30T+10C+100THU
10C+100THU
B
ns
ns
ns
***
ns
2.5
2.0
1.5
1.0
0.5
0.0
Relative RR1 mRNA expression
ns
ns
ns
***
ns
Relative RR2 mRNA expression
NT
10T
30T
10T+10C+100THU
30T+10C+100THU
10C+100THU

Apoptosis and Double-Stranded DNA breaks analyses following thymidine treatment.

A) Early, late apoptosis and necrosis were analyzed by flow cytometry analysis at 18 hours following treatment of HeLa cells with 10T, 30T, 10T+10C+100THU, 30T+10C+100THU and 10C+100THU. B) Relative expression of *RR1* and *RR2* subunits following treatment of HeLa cells with 10T, 30T, 10T+10C+100THU, 30T+10C+100THU and 10C+100THU. Gene expression was normalized using the expression level of the housekeeping gene *RPL13A*. C) Flow cytometry analysis of the γ-H2AX positive cells at 18 hours following treatment of HeLa cells with 10T, 30T, 10T+10C+100THU; 30T+10C+100THU and 10C+100THU. Actinomycin concentration at 0.5µM and 5µM was used as positive controls. For apoptosis and γ-H2AX analyses, mean values and standard error of the mean (s.e.m.) were calculated for three independent treatments ($n = 3$), Data were subjected to two-way ANOVA, followed by a Sidak post hoc test, *, $p < 0.05$, ** $p < .01$, ***, $p < .001$, ****, $p <.0001$, ns: not statistically significant.
