# Supplementary material for: Disruption of deoxyribonucleotide triphosphate biosynthesis leads to RAS proto-oncogene activation and perturbation of mitochondrial metabolism
Source: J Biol Chem. 2024 Dec 23;301(2):108117. doi: 10.1016/j.jbc.2024.108117 (PMC11791277; doi:10.1016/j.jbc.2024.108117)
Supplement: Supporting Figure S2 [file mmc2.pdf]

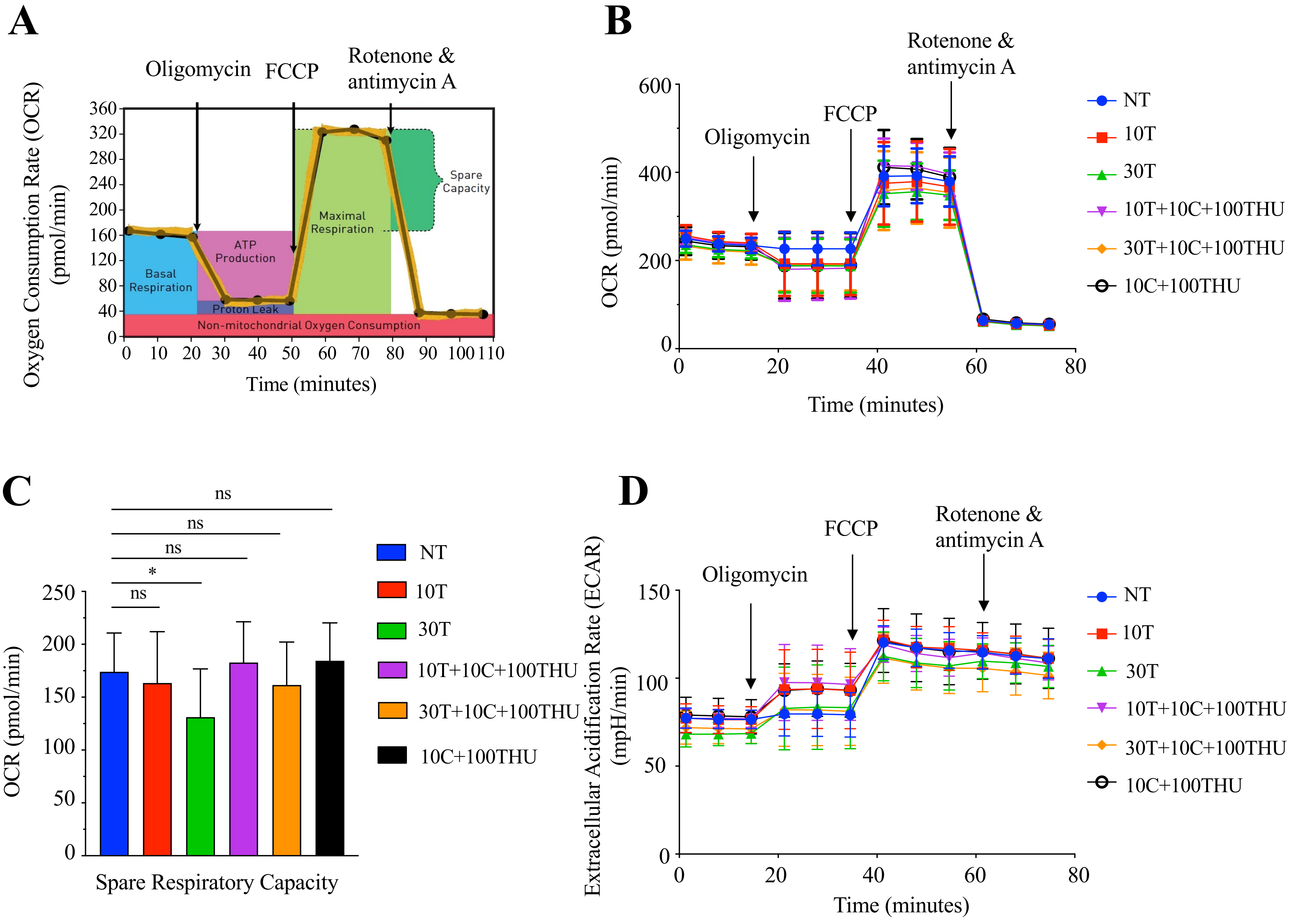
A
Oligomycin
FCCP
Rotenone & antimycin A
Oxygen Consumption Rate (OCR) (pmol/min)
Time (minutes)
Basal Respiration
ATP Production
Proton Leak
Maximal Respiration
Spare Capacity
Non-mitochondrial Oxygen Consumption
B
OCR (pmol/min)
Time (minutes)
NT
10T
30T
10T+10C+100THU
30T+10C+100THU
10C+100THU
C
ns
ns
ns
*
ns
OCR (pmol/min)
Spare Respiratory Capacity
D
Extracellular Acidification Rate (ECAR) (mpH/min)
Time (minutes)

Analysis of mitochondrial activity by the Seahorse extracellular flux analyzer.

A) Measure of the oxygen consumption rate (OCR, pMoles/min), indicative of OXPHOS in HeLa cells treated with 10T, 30T, 10T+10C+100THU, 30T+10C+100THU and 10C+100THU and NT cells used as control. After establishing a baseline, oligomycin (2μM), FCCP (0.8μM), and rotenone (0.5μM) were sequentially added. B) The OCR was measured at 18 hours under basal and treated conditions using Seahorse technology with a Mito stress test kit. Sequential injection of oligomycin (Oligom.), FCCP, and rotenone/antimycin A (Rot./Ant.) is indicated, mean values and s.e.m. were calculated for three independent experiments in duplicate (n=6). C) The spare respiratory capacity analysis in NT and treated conditions. Data from were subjected to two-way ANOVA, followed by a Sidak post hoc test, *, $p < 0.05$. D) Profiling of ECAR (mpH/min) in control and treated cells was measured in the same experiments as described in A and B, mean values and s.e.m. were calculated for three independent experiments in duplicate (n=6).
