# Supplementary material for: Disruption of deoxyribonucleotide triphosphate biosynthesis leads to RAS proto-oncogene activation and perturbation of mitochondrial metabolism
Source: J Biol Chem. 2024 Dec 23;301(2):108117. doi: 10.1016/j.jbc.2024.108117 (PMC11791277; doi:10.1016/j.jbc.2024.108117)
Supplement: Supporting Figure S3 [file mmc3.pdf]

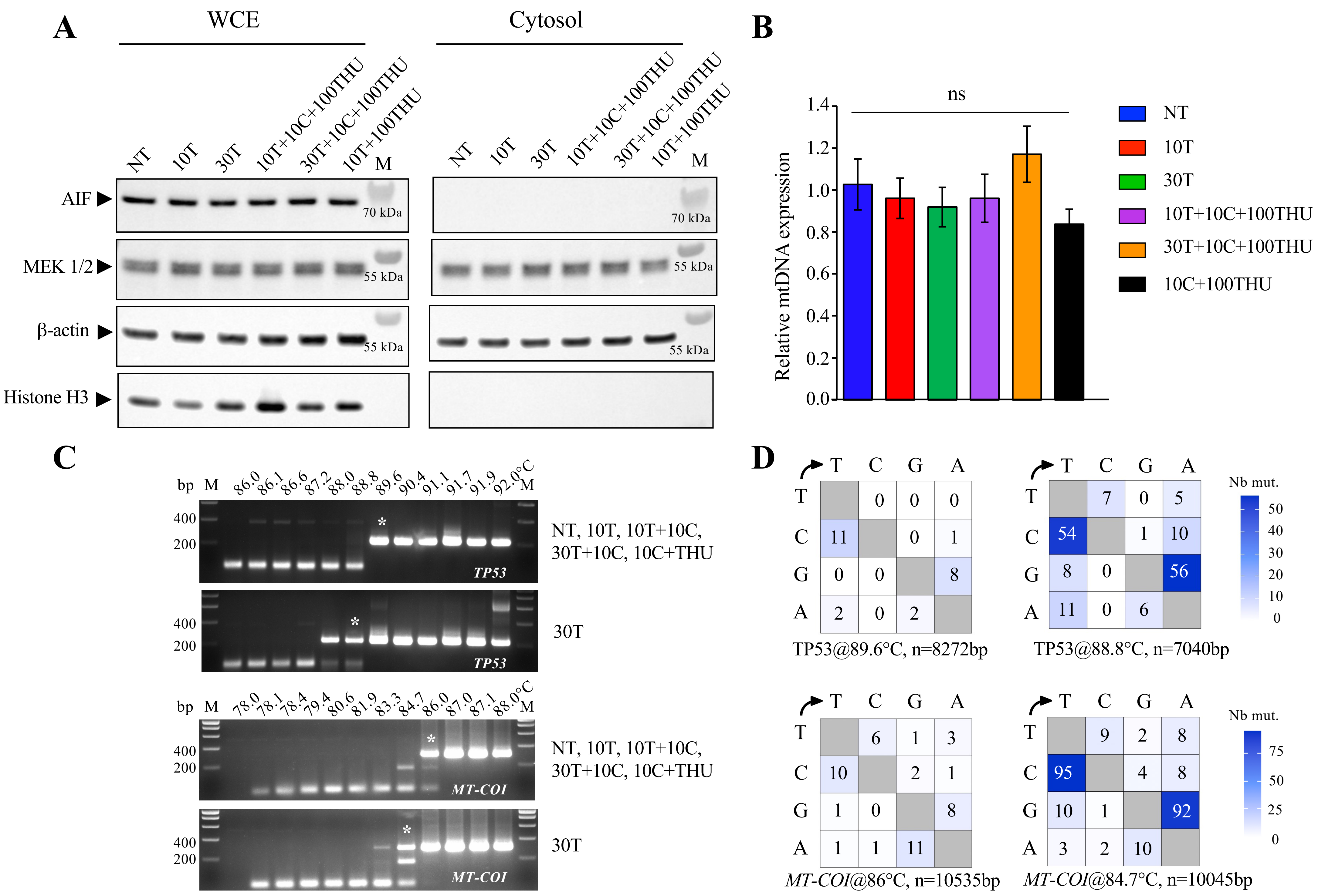

A
WCE
Cytosol
NT
10T
30T
10T+10C+100THU
30T+10C+100THU
10T+100THU
M
AIF
MEK 1/2
β-actin
Histone H3
70 kDa
55 kDa
B
ns
Relative mtDNA expression
1.4
1.2
1.0
0.8
0.6
0.4
0.2
0.0
NT
10T
30T
10T+10C+100THU
30T+10C+100THU
10C+100THU
C
bp
M
86.0 86.1 86.6 87.2 88.0 88.8 89.6 90.4 91.1 91.7 91.9 92.0°C
400
200
TP53
NT, 10T, 10T+10C, 30T+10C, 10C+THU
30T
78.0 78.1 78.4 79.4 80.6 81.9 83.3 84.7 86.0 87.0 87.1 88.0°C
MT-COI
D
T C G A
Nb mut.
TP53@89.6°C, n=8272bp
TP53@88.8°C, n=7040bp
MT-COI@86°C, n=10535bp
MT-COI@84.7°C, n=10045bp

Purity control of whole cell extract and cytosol by immunoblotting, mtDNA quantification and 3DPCR on *TP53* and *MT-COI*

A) Purity control of cellular fractions by immunoblotting, following treatment of HeLa cells, using β-actin and MEK1/2 as a marker of cytosolic proteins, AIF (apoptosis inducing factor) for mitochondria and Histone H3 as a marker of nuclear proteins. bp: base pairs, WCE, whole cell extract. B) Relative mtDNA production in whole cell extract (WCE) as a function of different treatments. C) 3DPCR analysis of *TP53* and *MT-COI* fragments, showing denaturation temperature as low as 88°C and 83.3°C respectively following different treatments. Asterisks indicate samples that were cloned and sequenced. D) Mutation matrices derived from cloned 3DPCR products obtained at 89.6°C and 88.8°C for *TP53* and 86°C and 84.7°C for *MT-COI* sequences . n indicates the total number of bases sequenced.
