# Supplementary material for: Disruption of deoxyribonucleotide triphosphate biosynthesis leads to RAS proto-oncogene activation and perturbation of mitochondrial metabolism
Source: J Biol Chem. 2024 Dec 23;301(2):108117. doi: 10.1016/j.jbc.2024.108117 (PMC11791277; doi:10.1016/j.jbc.2024.108117)
Supplement: Supporting Figure S4 [file mmc4.pdf]

```
KRAS      ATGACTGAATATAAACTTGTGGTAGTTGGAGCTGGTGGCGTAGGCAAGAGTGCCTTGACG    60
HRAS      ATGACGGAATATAAGCTGGTGGTGGTGGGCGCCGGCGGTGTGGGCAAGAGTGCGCTGACC    60
          ***** ******** ** ***** ** ** ** ** ** ** ***********  ****

KRAS      ATACAGCTAATTCAGAATCATTTTGTGGACGAATATGATCCAACAATAGAGGATTCCTAC    120
HRAS      ATCCAGCTGATCCAGAACCATTTTGTGGACGAATACGACCCCACTATAGAGGATTCCTAC    120
          ** ***** ** ***** ***************** ** ** ** ***************

KRAS      AGGAAGCAAGTAGTAATTGATGGAGAAACCTGTCTCTTGGATATTCTCGACACAGCAGGT    180
HRAS      CGGAAGCAGGTGGTCATTGATGGGGAGACGTGCCTGTTGGACATCCTGGATACCGCCGGC    180
           ******* ** ** ******** ** ** ** ** ***** ** ** ** ** ** **

KRAS      CAAGAGGAGTACAGTGCAATGAGGGACCAGTACATGAGGACTGGGGAGGGCTTTCTTTGT    240
HRAS      CAGGAGGAGTACAGCGCCATGCGGGACCAGTACATGCGCACCGGGGAGGGCTTCCTGTGT    240
          ** *********** ** *** ************** * ** *********** ** ***

KRAS      GTATTTGCCATAAATAATACTAAATCATTTGAAGATATTCACCATTATAGAGAACAAATT    300
HRAS      GTGTTTGCCATCAACAACACCAAGTCTTTTGAGGACATCCACCAGTACAGGGAGCAGATC    300
          ** ******** ** ** ** ** ** ***** ** ** ***** ** ** ** ** **

KRAS      AAAAGAGTTAAGGACTCTGAAGATGTACCTATGGTCCTAGTAGGAAATAAATGTGATTTG    360
HRAS      AAACGGGTGAAGGACTCGGATGACGTGCCCATGGTGCTGGTGGGGAACAAGTGTGACCTG    360
          *** * ** ******** ** ** ** ** ***** ** ** ** ** ** *****  **

KRAS      CCTTCTAGAACAGTAGACACAAAACAGGCTCAGGACTTAGCAAGAAGTTATGGAATTCCT    420
HRAS      GCTGCACGCACTGTGGAATCTCGGCAGGCTCAGGACCTCGCCCGAAGCTACGGCATCCCC    420
           ** *  * ** ** **  *    ************ * **  **** ** ** ** **

KRAS      TTTATTGAAACATCAGCAAAGACAAGACAGGGTGTTGATGATGCCTTCTATACATTAGTT    480
HRAS      TACATCGAGACCTCGGCCAAGACCCGGCAGGGAGTGGAGGATGCCTTCTACACGTTGGTG    480
          *  ** ** ** ** ** *****  * ***** ** ** *********** ** ** **

KRAS      CGAGAAATTCGAAAACATAAAGAAAAGATGAGCAAAGATGGTAAAAAGAAGAAAAAGAAG    540
HRAS      CGTGAGATCCGGCAGCACAAGCTGCGGAAGCTGAACCCTCCTGATGAGAGTGGCCCCGGC    540
          ** ** ** **  * ** **      ** *   **   *  * *  ***

KRAS      TCAAAGA---CAAAGTGTGTAATTATGTAA    567
HRAS      TGCATGAGCTGCAAGTGTGTGCTCTCCTGA    570
          *  * **     ******** *    * *
```

CG->TA   TA->CG   Neutral mutations

Alignment of the *HRAS* and *KRAS* genes and comparison of the of GC base content.

HRAS is used as reference, green rectangles: CG->AT mutation, red rectangles: AT->GC mutations, blue rectangle: neutral mutations.
