# Supplementary material for: Disruption of deoxyribonucleotide triphosphate biosynthesis leads to RAS proto-oncogene activation and perturbation of mitochondrial metabolism
Source: J Biol Chem. 2024 Dec 23;301(2):108117. doi: 10.1016/j.jbc.2024.108117 (PMC11791277; doi:10.1016/j.jbc.2024.108117)
Supplement: Supporting Figure S5 [file mmc5.pdf]

A

Mutated *KRAS*/Tumor

08 09 10 11 12 13 14 15 16 17<br>V V G A G G V G K S<br>GTAGTTGGAGCT**GGTGGC**GTAGGCAAGAGT		
`.............A................`	04, 33, 66, 71, 80, 84, 101, 130	G12D
`.......T.....A................`	14	G12D
`.............A............T...`	97	G12D
`.............T................`	09, 11, 51, 69, 105	G12V
`........T....T................`	63	G12V
`........T....T...........T.T..`	86	G12V
`.............C................`	06, 23, 46, 61, 74, 102, 125	G12A
`........T....C................`	64	G12A
`............T.................`	07, 38, 115, 135	G12C
`............A.................`	08	G12S
`............C.................`	13, 120	G12R
`................A.............`	29, 31, 49, 134	G13D
`.......T........A.........T...`	50	G13D
`.......T........A........T.T..`	60	G13D
`..............A.A.............`	70	G13D

WT *KRAS*/Tumor

08 09 10 11 12 13 14 15 16 17<br>V V G A G G V G K S<br>GTAGTTGGAGCT**GGTGGC**GTAGGCAAGAGT	
`........T.....................`	53
`..........................T...`	22, 26, 72
`.......T..................T...`	12, 89, 106, 116, 127, 131
`...........................T..`	41, 43
`........T...............TT....`	55

B

Mutated *KRAS*/Healthy tissue

08 09 10 11 12 13 14 15 16 17<br>V V G A G G V G K S<br>GTAGTTGGAGCT**GGTGGC**GTAGGCAAGAGT	
`........T.....................`	66, 80
`.......T..................T...`	11, 29
`........T..................T..`	60, 61
`........T................T.T..`	63, 71, 86
`T......T..................T...`	134

WT *KRAS*/Healthy tissue

08 09 10 11 12 13 14 15 16 17<br>V V G A G G V G K S<br>GTAGTTGGAGCT**GGTGGC**GTAGGCAAGAGT	
`........T.....................`	77, 83, 87
`.......T......................`	128
`........T................T....`	62

Mutations in the *KRAS* gene in tumours and healthy tissues.
A) Mutations in the *KRAS* gene in tumours. B) Mutations in the *KRAS* gene in healthy tissues. Mutational hotspots are located in amino acids Gly_{12} and Gly_{13} and are highlighted in red. The numbers represent the sequences containing mutations. The remaining not represented sequences are non-mutated.
