# Supplementary material for: Disruption of deoxyribonucleotide triphosphate biosynthesis leads to RAS proto-oncogene activation and perturbation of mitochondrial metabolism
Source: J Biol Chem. 2024 Dec 23;301(2):108117. doi: 10.1016/j.jbc.2024.108117 (PMC11791277; doi:10.1016/j.jbc.2024.108117)
Supplement: Supporting Table S1 [file mmc6.docx]

| **Patients** | **Age** | **Sex** | **Tumor localization**  **in colon** | **Stage** | **TNIM^a^** | **pTNM^b^** | **CD3^+^** | **CD8^+^** | **CD45**  **RO^+^** | **Diff.** | **Vascular invasion** | **%**  **tumoral cells** | **MSI status** | **KRAS** | **BRAF** | **PI3K** | **MLH1** | **HERV** | **LNE-1** |
| --- | --- | --- | --- | --- | --- | --- | --- | --- | --- | --- | --- | --- | --- | --- | --- | --- | --- | --- | --- |
| 1 | 70 | M | Left | 2 | T3N0M0 | T3N0 | +/- | + | + | Moderate | No | 30 | MSS | - | - | - | - | + | + |
| 2 | 74 | M | Sigmoid | 1 | T2N0M0 | T2N0 | 0 | 0 | 0 | Few | No | 60 | MSS | - | - | - | - | + | + |
| 3 | 93 | M | Right | 2 | T4N0M0 | T4aN0 | + | + | + | Few | Yes | 80 | MSI-H | - | + | - | + | + | + |
| 4 | 75 | M | Left | 4 | T3N2M1 | T3N1b | 0 | 0 | 0 | Moderate | Yes | 40 | MSS | G12D | - | - | - | - | + |
| 5 | 95 | F | Left | 2 | T4N0M0 | T4aN0 | 0 | 0 | 0 | Few | No | 40 | MSI-H | - | + | + | + | + | + |
| 6 | 87 | M | Sigmoid | 2 | T3N0M0 | T3N0 | 0 | 0 | +/- | Moderate | No | 80 | MSS | G12A | - | - | - | + | + |
| 7 | 84 | F | Right | 3 | T3N1M0 | T3N1b |  | + | + | Moderate | No | 30 | MSS | G12C | + | - | - | - | + |
| 8 | 58 | F | Right | 2 | T3N0M0 | T3N0 | 0 | 0 | 0 | Moderate | No | 30 | MSS | G12S | - | - | - | - | + |
| 9 | 68 | M | Sigmoid | 3 | T4N2M0 | T4aN2a | + | + | 0 | Few | Yes | 40 | MSS | G12V | - | - | - | - | + |
| 10 | 72 | M | Sigmoid | 2 | T3N0M0 | T3N0 | + | + | +/- | Moderate | No | 70 | MSS | - | - | - | - | + | + |
| 11 | 62 | M | Right | 3 | T3N2M0 | T3N2b | 0 | 0 | 0 | Few | Yes | 90 | MSS | G12V | - | - | - | + | + |
| 12 | 73 | M | Left | 2 | T3N0M0 | T3N0 | 0 | 0 | 0 | Moderate | No | 20 | MSS | - | - | - | - | - | + |
| 13 | 89 | F | Right | 2 | T3N0M0 | T3N0 | + | + | + | Few | No | 80 | MSI-H | G12R | - | - | + | + | + |
| 14 | 48 | F | Sigmoid | 2 | T3N0M0 | T3N0 | 0 | 0 | 0 | Moderate | No | 30 | MSS | G12D | - | - | - | - | + |
| 15 | 75 | M | Left colon | 3 | T3N1M0 | T3N1a | 0 | 0 | 0 | Moderate | No | 60 | MSS | - | - | - | - | + | + |
| 16 | 69 | F | Right | 2 | T3N0M0 | T3N0 | + | + | +/- | Few | No | 70 | MSI-H | - | + | - | + | + | + |
| 17 | 88 | F | Right | 2 | T3N0M0 | T3N0 | 0 | 0 | + | Moderate | Yes | 80 | MSI-H | - | + | - | - | + | + |
| 18 | 76 | M | Right | 3 | T4N1M0 | T4aN1b | + | + | + | Few | Yes | 60 | MSI-H | - | + | - | - | - | + |
| 19 | 73 | F | Right | 3 | T3N1M0 | T3N1b | + | + | + | Few | No | ? | MSI-H | - | + | - | + | + | + |
| 20 | 67 | F | Sigmoid | 2 | T3N0M0 | T3N0 | + | + | +/- | Moderate | No | 80 | MSS | - | - | - | - | - | + |
| 21 | 74 | M | Left | 3 | T3N1M0 | T3N1a | 0 | 0 | 0 | Moderate | No | 70 | MSS | - | - | - | - | - | + |
| 22 | 69 | M | Left | 2 | T4N0M0 | T4aN0 | + | + | + | Moderate | No | ? | MSS | - | - | - | - | + | + |
| 23 | 88 | M | Sigmoid | 1 | T2N0M0 | T2N0 | 0 | 0 | 0 | Moderate | No | 70 | MSS | G12A | - | - | - | + | + |
| 24 | 71 | F | Sigmoid | 3 | T3N2M0 | T3N2b | 0 | 0 | 0 | Moderate | Yes | 80 | MSS | - | - | - | - | - | + |
| 25 | 66 | M | Right | 2 | T3N0M0 | T3N0 | + | + | + | Few | Yes | 90 | MSS | - | - | - | - | - | + |
| 26 | 88 | M | Right | 4 | T4N2M0 | T4aN2b | 0 | 0 | 0 | Few | Yes | 80 | MSS | - | - | - | - | - | + |
| 27 | 87 | F | Right | 2 | T3N0M0 | T3N0 | + | + | 0 | Moderate | Yes | 50 | MSI-H | - | + | - | + | + | + |
| 28 | 76 | F | Sigmoid | 2 | T4N0M0 | T4bN0 | +/- | 0 | 0 | Moderate | No | 50 | MSS | - | - | - | - | + | + |
| 29 | 79 | F | Right | 4 | T3N0M1 | T3N0M1a | 0 | 0 | 0 | Few | Yes | 80 | MSS | G13D | - | + | - | + | - |
| 30 | 56 | M | Sigmoid | 2 | T3N0M0 | T3N0 | 0 | 0 | 0 | Moderate | Yes | 80 | MSS | - | - | - | - | + | + |
| 31 | 75 | M | Left | 3 | T3N1M0 | T3N1a | +/- | +/- | 0 | Moderate | Yes | ? | MSS | G13D | - | - | - | - | + |
| 32 | 62 | M | Sigmoid | 1 | T2N0M0 | T2N0 | 0 | 0 | 0 | Adequate | No | 70 | MSS | - | - | - | - | + | + |
| 33 | 68 | M | Left | 2 | T3N0M0 | T3N0 | 0 | 0 | 0 | Moderate | No | 80 | MSS | G12D | - | - | - | + | + |
| 34 | 74 | M | Right | 2 | T3N0M0 | T3N0 | 0 | 0 | 0 | Moderate | No | 30 | MSS | - | + | - | - | + | + |
| 35 | 63 | F | Right | 2 | T3N0M0 | T3N0 | +/- | + | +/- | Moderate | No | 10 | MSS | - | - | + | - | + | + |
| 36 | 61 | F | Right | 3 | T3N2M0 | T3N1b | 0 | 0 | +/- | Moderate | Yes | ? | MSS | - | - | - | - | - | + |
| 37 | 67 | F | Sigmoid | 4 | T4N0M1 | T4aN0 | 0 | 0 | 0 | Moderate | Yes | ? | MSS | - | - | - | - | - | ? |
| 38 | 94 | M | Right | 3 | T4N1M0 | T4aN1a | 0 | 0 | 0 | Moderate | No | 90 | MSS | G12C | - | - | - | + | + |
| 39 | 64 | M | Sigmoid | 1 | T2N0M0 | T2N0 | +/- | + | + | Adequate | No | 80 | MSS | - | - | - | - | - | - |
| 40 | 61 | M | Right | 4 | T4N0M0 | T4aN0M1b | + | +/- | + | Few | No | ? | MSS | - | - | - | - | - | + |
| 41 | 78 | M | Left | 4 | T3N2M1 | T3N2aM1b | 0 | 0 | 0 | Few | Yes | 50 | MSS | - | - | - | - | - | + |
| 42 | 80 | F | Right | 3 | T3N1M0 | T3N1a | 0 | 0 | 0 | Moderate | Yes | 50 | MSS | - | - | - | - | + | + |
| 43 | 81 | F | Sigmoid | 1 | T2N0M0 | T2N0 | 0 | 0 | 0 | Moderate | No | 70 | MSS | -. | - | - | - | - | - |
| 44 | 93 | F | Right | 2 | T3N0M0 | T3N0 | + | + | 0 | Moderate | No | 40 | MSI-H | - | - | - | + | + | + |
| 45 | 65 | M | Left | 4 | T3N0M1 | T3N0 | 0 | 0 | 0 | Moderate | No | 20 | MSS | - | - | + | - | + | + |
| 46 | 52 | F | Sigmoid | 3 | T4N1M0 | T3N1a | 0 | 0 | +/- | Moderate | Yes | 70 | MSS | G12A | - | - | - | + | + |
| 47 | 82 | F | Right | 4 | T4N0M1 | T4aN0M1a | + | + | 0 | Moderate | Yes | 50 | MSI-L | - | + | - | + | + | + |
| 48 | 59 | M | Sigmoid | 1 | T2N0M0 | T2N0 | +/- | + | + | Moderate | No | ? | MSS | - | - | - | - | + | + |
| 49 | 83 | F | Right | 3 | T4N1M0 | T4aN1b | +/- | +/- | +/- | Moderate | No | 20 | MSI-H | G13D | - | + | - | + | + |
| 50 | 70 | F | Left | 2 | T4N0M0 | T4bN0 | 0 | 0 | 0 | Moderate | No | 80 | MSS | G13D | - | - | - | + | ? |
| 51 | 49 | F | Left | 4 | T4N1M1 | T4N1b | 0 | 0 | 0 | Moderate | Yes | 0 | MSS | G12V | - | - | - | - | + |
| 52 | 53 | M | Sigmoid | 3 | T2N1M0 | T2N1b | 0 | 0 | 0 | Moderate | No | 70 | MSS | - | - | - | - | + | ? |
| 53 | 78 | M | Right | 4 | T3N2M1 | T3N2b | +/- | +/- | 0 | Moderate | Yes | 90 | MSS | - | - | - | - | - | - |
| 54 | 64 | M | Left | 2 | T3N0M0 | T3N0 | 0 | 0 | 0 | Adequate | No | 90 | MSS | - | - | - | - | - | ? |
| 55 | 57 | F | Sigmoid | 2 | T3N0M0 | T3N0 | +/- | + | 0 | Moderate | No | 90 | MSS | - | - | - | - | + | + |
| 56 | 55 | F | Right | 2 | T3N0M0 | T3N0 | + | + | 0 | Moderate | No | 40 | MSS | - | - | - | - | - | + |
| 57 | 75 | F | Sigmoid | 2 | T3N0M0 | T2N0 | + | + | + | Moderate | No | ? | MSS | - | - | - | - | + | + |
| 58 | 60 | M | Left | 3 | T3N2M0 | T3N2b | + | + | +/- | Adequate | Yes | 80 | MSS | - | - | - | - | + | + |
| 59 | 68 | M | Right | 2 | T3N0M0 | T3N0 | + | + | + | Few | No | 90 | MSI-H | - | + | - | + | + | + |
| 60 | 75 | M | Right | 4 | T4N1M1 | T3N1bM1a | 0 | 0 | 0 | Moderate | Yes | 90 | MSS | G13D | - | - | - | - | + |
| 61 | 50 | M | Right | 4 | T4N1M1 | T4aN1bM1a | 0 | 0 | 0 | Adequate | Yes | 80 | MSS | G12A | - | + | - | - | ? |
| 62 | 71 | F | Left | 4 | T3N0M1 | T3N0 | 0 | +/- | 0 | Few | Yes | 90 | MSS | - | - | + | - | - | + |
| 63 | 83 | M | Right | 4 | T3N0M1 | T3N0M1a | 0 | 0 | 0 | Moderate | Yes | 40 | MSS | G12V | - | - | - | - | - |
| 64 | 74 | F | Right | 3 | T4N2M1 | T4aN2a | 0 | 0 | 0 | Adequate | Yes | 70 | MSS | G12A | - | - | - | + | + |
| 65 | 93 | F | Sigmoid | 3 | T3N2M0 | T3N2a | + | + | + | Adequate | Yes | 45 | MSS | - | - | - | - | + | + |
| 66 | 85 | M | Sigmoid | 2 | T3N0M0 | T3N0 | 0 | 0 | 0 | Moderate | No | 50 | MSS | G12D | - | - | - | + | + |
| 67 | 60 | F | Left | 3 | T3N1M0 | T3N1a | 0 | 0 | 0 | Moderate | Yes | 30 | MSS | - | - | - | - | + | + |
| 68 | 73 | M | Left | 2 | T3N0M0 | T3N0 | + | + | + | Moderate | No | 60 | MSS | - | - | - | - | + | ? |
| 69 | 51 | M | Sigmoid | 2 | T4N0M0 | T4bN0 | + | + | + | Few | Yes | 80 | MSI-H | G12V | - | + | - | + | + |
| 70 | 75 | F | Left | 3 | T3N1M0 | T3N1a | 0 | 0 | + | Few | No | 70 | MSS | G13D | - | - | - | + | + |
| 71 | 74 | F | Sigmoid | 3 | T3/4N1M0 | T3N1b | 0 | 0 | 0 | Moderate | No | 70 | MSS | G12D | - | + | - | + | + |
| 72 | 73 | M | Sigmoid | 2 | T4N0M0 | T4aN0 | 0 | 0 | +/- | Moderate | No | 70 | MSS | - | - | - | - | + | + |
| 73 | 62 | M | Right | 3 | T4N2M0 | T4aN2a | +/- | +/- | 0 | Moderate | Yes | 20 | MSS | - | + | - | - | + | + |
| 74 | 36 | F | Left | 3 | T3N1M0 | T3N1b | 0 | 0 | 0 | Moderate | Yes | 30 | MSI-H | G12A | - | + | - | + | + |
| 75 | 74 | M | Sigmoid | 4 | T4N2M1 | T4aN2bM1b | 0 | 0 | 0 | Few | Yes | 60 | MSS | - | - | - | - | + | + |
| 76 | 58 | M | Right | 1 | T2N0M0 | T2N0 | + | + | + | Moderate | No | 80 | MSS | - | - | - | - | + | + |
| 77 | 72 | F | Right | 2 | T3N0M0 | T3N0 | +/- | + | + | Moderate | No | 70 | MSI-H | - | - | - | + | + | + |
| 78 | 88 | F | Right | 2 | T2N1M0 | T2N1b | + | + | + | Few | No | 25 | MSI-H | - | + | - | + | + | + |
| 79 | 85 | M | Left | 3 | T3N1M0 | T3N1b | 0 | 0 | 0 | Few | Yes | 60 | MSS | - | - | - | - | + | + |
| 80 | 55 | M | Right | 4 | T4N2M1 | T4aN2aM1b | 0 | 0 | 0 | Few | Yes | 80 | MSS | G12D | - | - | - | + | + |
| 81 | 89 | F | Right | 3 | T3N1M0 | T3N1b | + | + | + | Few | Yes | 55 | MSI-H | - | + | + | + | + | + |
| 82 | 59 | M | Right | 3 | T4N1M0 | T4aN1a | 0 | 0 | 0 | Adequate | Yes | 65 | MSS | - | - | - | - | + | ? |
| 83 | 63 | M | Sigmoid | 1 | T2N0M0 | T2N0 | 0 | 0 | 0 | Moderate | Yes | 30 | MSS | - | - | - | - | + | + |
| 84 | 45 | F | Left | 3 | T4N1M0 | T4aN1a | 0 | 0 | 0 | Moderate | No | ? | MSS | G12D | - | - | - | + | + |
| 85 | 74 | F | Left | 2 | T4N0M0 | T4bN0 | + | + | + | Moderate | No | 80 | MSS | - | - | - | - | - | - |
| 86 | 55 | M | Left | 3 | T4N2M1 | T4bN2a | 0 | 0 | 0 | Moderate | Yes | ? | MSS | G12V | - | - | - | + | + |
| 87 | 61 | F | Sigmoid | 2 | T3N0M0 | T3N0 | 0 | 0 | 0 | Moderate | Yes | 40 | MSS | - | - | - | - | + | + |
| 88 | 60 | F | Left | 4 | T4N0M1 | T4aN0M1a | 0 | 0 | 0 | Moderate | Yes | 30 | MSS | - | - | - | - | + | + |
| 89 | 70 | M | Sigmoid | 1 | T2N0M0 | T2N0 | 0 | 0 | 0 | Moderate | No | 20 | MSS | - | - | - | - | + | + |
| 90 | 69 | M | Sigmoid | 3 | T2N1M0 | T2N1a | 0 | 0 | +/- | Moderate | No | 80 | MSS | - | - | - | - | + | + |
| 91 | 76 | F | Sigmoid | 4 | T4N0M1 | T4bN0 | 0 | 0 | 0 | Moderate | No | 50 | MSS | - | - | - | - | + | + |
| 92 | 45 | M | Right | 4 | T4N1M1 | T4aN1bM1b | 0 | 0 | 0 | Few | Yes | 80 | MSS | - | - | - | - | - | + |
| 93 | 53 | M | Sigmoid | 3 | T3N2M0 | T3N2a | 0 | 0 | + | Moderate | Yes | ? | MSS | - | - | - | - | + | + |
| 94 | 90 | F | Sigmoid | 2 | T3N0M0 | T3N0 | 0 | 0 | 0 | Few | No | 70 | MSI-H | - | - | + | - | + | + |
| 95 | 80 | F | Right | 3 | T4N2M0 | T4aN2a | 0 | 0 | 0 | Moderate | Yes | 0 | MSS | - | - | - | - | - | - |
| 96 | 85 | M | Left | 4 | T3N0M1 | T3N0 | 0 | 0 | 0 | Adequate | No | ? | MSS | - | - | - | - | - | - |
| 97 | 61 | M | Right | 4 | T4N1M1 | T4aN1aM1a | 0 | 0 | 0 | Adequate | Yes | 50 | MSS | G12D | - | - | - | - | + |
| 98 | 84 | M | Sigmoid | 2 | T3N0M0 | T3N0 | 0 | 0 | 0 | Adequate | No | 70 | MSS | - | - | - | - | - | + |
| 99 | 73 | M | Sigmoid | 1 | T2N0M0 | T2N0 | 0 | 0 | +/- | Moderate | No | ? | MSS | - | - | - | - | + | + |
| 100 | 74 | F | Right | 4 | T4N2M1 | T4aN2bM1b | 0 | 0 | +/- | Few | Yes | 50 | MSI-H | - | - | + | + | + | + |
| 101 | 73 | M | Sigmoid | 2 | T3N0M0 | T3N0 | 0 | 0 | 0 | Moderate | No | 60 | MSS | G12D | - | - | - | + | + |
| 102 | 87 | F | Right | 4 | T4N2M1 | T4aN2bM1b | 0 | 0 | 0 | Moderate | Yes | 70 | MSS | G12A | - | - | - | ? | + |
| 103 | 74 | F | Left | 3 | T4N1M0 | T4aN1a | 0 | +/- | 0 | Moderate | No | 50 | MSS | - | + | - | - | + | + |
| 104 | 64 | F | Right | 3 | T3N1M0 | T3N1a | 0 | 0 | 0 | Moderate | Yes | 60 | MSS | - | - | - | - | ? | + |
| 105 | 86 | F | Left | 3 | T4N1M0 | T4aN1a | +/- | 0 | 0 | Moderate | No | ? | MSS | G12V | - | - | - | - | + |
| 106 | 82 | M | Sigmoid | 2 | T3N0M0 | T3N0 | 0 | 0 | 0 | Moderate | No | 40 | MSS | - | - | - | - | + | + |
| 107 | 78 | M | Sigmoid | 4 | T4N0M1 | T4aN0 | 0 | 0 | 0 | Moderate | No | ? | MSS | - | - | - | - | + | + |
| 108 | 83 | M | Right | 2 | T3N0M0 | T3N0 | + | + | +/- | Adequate | No | 90 | MSI-H | - | + | - | + | + | + |
| 109 | 70 | F | Left | 4 | T4N2M1 | T4aN2a | 0 | 0 | 0 | Moderate | Yes | 25 | MSS | - | + | + | - | + | + |
| 110 | 70 | M | Right | 4 | T4N2M1 | T4aN2bM1b | 0 | 0 | 0 | Moderate | Yes | 20 | MSS | - | - | + | - | + | + |
| 111 | 65 | F | Sigmoid | 2 | T4N0M0 | T4N0 | 0 | 0 | 0 | Moderate | No | 40 | MSS | - | - | - | - | + | + |
| 112 | 73 | F | Left | 3 | T4N1M0 | T4aN1a | +/- | + | +/- | Moderate | No | 80 | MSS | - | - | - | - | - | + |
| 113 | 57 | F | Left | 2 | T3N0M0 | T3N0 | 0 | 0 | 0 | Moderate | No | 50 | MSS | - | - | - | - | ? | + |
| 114 | 90 | F | Left | 2 | T4N0M0 | T4aN0 | 0 | 0 | 0 | Adequate | No | 25 | MSS | - | - | - | - | ? | + |
| 115 | 78 | M | Right | 3 | T3N1M0 | T3N1a | + | 0 | +/- | Moderate | Yes | 60 | MSS | G12C. | - | - | - | ? | + |
| 116 | 72 | F | Left | 3 | T3N1M0 | T3N1b | 0 | 0 | 0 | Moderate | No | 30 | MSS | - | + | - | - | ? | + |
| 117 | 82 | M | Sigmoid | 3 | T3N1M0 | T3N1a | 0 | 0 | +/- | Moderate | No | 60 | MSS | - | - | - | - | ? | + |
| 118 | 83 | M | Right | 2 | T3N0M0 | T3N0 | +/- | + | + | Moderate | No | ? | MSS | - | - | - | - | - | + |
| 119 | 88 | F | Right | 3 | T4N1M0 | T4aN2a | 0 | 0 | 0 | Few | No | 80 | MSI-H | - | + | - | - | - | + |
| 120 | 69 | F | Right | 2 | T3N0M0 | T3N0 | 0 | 0 | 0 | Moderate | No | 70 | MSS | G12R | - | - | - | - | + |
| 121 | 38 | F | Sigmoid | 1 | T2N0M0 | T2N0 | + | + | + | Moderate | No | ? | MSS | - | - | - | - | ? | ? |
| 122 | 75 | M | Left | 2 | T3N0M0 | T3N0 | 0 | 0 | 0 | Moderate | No | 45 | MSS | - | - | - | - | + | + |
| 123 | 72 | F | Right | 4 | T4N1M1 | T4aN1b | 0 | 0 | 0 | Moderate | Yes | 70 | MSI-H | - | - | - | + | - | + |
| 124 | 84 | M | Left | 2 | T3N0M0 | T3N0 | 0 | 0 | 0 | Moderate | No | 30 | MSS | - | - | - | - | - | + |
| 125 | 78 | F | Sigmoid | 2 | T3N0M0 | T3N0 | 0 | 0 | 0 | Adequate | Yes | 80 | MSS | G12A | - | + | - | - | + |
| 126 | 74 | M | Sigmoid | 3 | T3N1M0 | T3N1b | 0 | 0 | 0 | Moderate | Yes | 70 | MSS | - | - | - | - | - | + |
| 127 | 78 | M | Right | 3 | T3N1M0 | T3N1a | + | + | + | Few | Yes | 85 | MSI-H | - | - | - | + | - | + |
| 128 | 73 | M | Left | 4 | T4N2M0 | T4aN2bM1b | 0 | 0 | 0 | Few | Yes | 60 | MSS | - | - | - | - | ? | + |
| 129 | 53 | M | Sigmoid | 4 | T3/4N2M0 | T4aN2a | 0 | 0 | 0 | Moderate | Yes | 25 | MSS | - | - | - | - | + | + |
| 130 | 81 | M | Right | 1 | T2N0M0 | T2N0 | 0 | 0 | +/- | Moderate | No | 30 | MSS | G12D | - | - | - | - | + |
| 131 | 68 | F | Left | 2 | T4N0M0 | T4aN0 | + | + | + | Moderate | No | 40 | MSS | - | - | - | - | + | ? |
| 132 | 83 | M | Right | 3 | T3N1M0 | T3N1b | + | + | 0 | Few | No | 90 | MSI-H | - | - | - | - | + | + |
| 133 | 85 | F | Right | 2 | T3N0M0 | T3N0 | 0 | 0 | 0 | Moderate | No | 40 | MSS | - | - | + | - | + | + |
| 134 | 74 | M | Sigmoid | 3 | T2N1M0 | T2N1a | +/- | +/- | 0 | Moderate | No | 90 | MSS | G13D | - | - | - | + | ? |
| 135 | 65 | M | Right colon | 4 | T4N1M1 | T4aN1bM1b | 0 | 0 | 0 | Few | Yes | 50 | MSS | G12C | - | - | - | + | ? |

Presentation of the 135 studied patients with colorectal cancers.
