# Supplementary material for: Disruption of deoxyribonucleotide triphosphate biosynthesis leads to RAS proto-oncogene activation and perturbation of mitochondrial metabolism
Source: J Biol Chem. 2024 Dec 23;301(2):108117. doi: 10.1016/j.jbc.2024.108117 (PMC11791277; doi:10.1016/j.jbc.2024.108117)
Supplement: Supporting Table S2 [file mmc7.docx]

**Primer Sequences**

____________________________________________________________________________________________________________________

*HRAS*ext fwd 5’TCGGCAGTACTCAGATCGGAAGAAATGACGGAATATAAGCTGGTG

*HRAS*ext rev 5’GGCGGTACCGACGTGCGTAGCTACCATGTCAGGAGAGCACACACTTGCAGCT

*HRAS*HindIII fwd 5’GGCAAGCTTTCGGCAGTACTCAGATCGGAAGAAATG

*HRAS*KpnI rev 5' GGCGGTACCGGCGGTACCGACGTGCGTAGCTACCATGTCA

*SEQRAS*mutf wd 5’TCGTCGGCAGCGTCAGATGTGTATAAGAGACAGTCGGCAGTACTCAGATCGGAAGAAATG

*SEQRAS*mut rev 5’GTCTCGTGGGCTCGGAGATGTGTATAAGAGACAGGGCGGTACCGACGTGCGTAGCTACCATGTCA

SEQRASdeep fwd 5’TCGTCGGCAGCGTCAGATGTGTATAAGAGACAGggcaggtggggcaggagaccctgta

SEQRASdeep rev 5’GTCTCGTGGGCTCGGAGATGTGTATAAGAGACAGCTGTGTCCTGGGCTCGCCCGCAGCA

*HRAS_A11V_* fwd 5’ aagctggtggtggtgggcgTcggcggtgtgggcaagagt

*HRAS_A11V_* rev 5’ ACTCTTGCCCACACCGCCGACGCCCACCACCACCAGCTT

*HRAS_G12C_* fwd 5’ctggtggtggtgggcgccTgcggtgtgggcaagagtGCG

*HRAS_G12C_* rev 5’CGCACTCTTGCCCACACCGCAGGCGCCCACCACCACCAG

*HRAS* SYBR fwd 5’ATGACGGAATATAAGCTGGTGGTGG

*HRAS* SYBR rev 5’GTTCTGGATCAGCTGGATGGTCAG

*KRAS* SYBR fwd 5’ATGACTGAATATAAACTTGTGG

*KRAS* SYBR rev 5'GAATTAGCTGTATCGTCAAGG

*dHCoxI* fwd 5’CCTCCCTTAGCAGGGAACTAC

*dHCoxI* rev 5’CACCTGCTAGGTGTAAGGAGAAG

*β2M* fwd 5’AATCAGATGGGTGTAGATCAAGG

*β2M* rev 5’GTTTCCACCCCTTCCATTTT

*RRM1* fwd 5’ CACCAGCAAAGATGAGGTTGC

*RRM1* rev 5’ GGGGCGATGGCGTTTATTTG

*RRM2* fwd 5′ TTTAGTGAGCTTAGCACAGCGGGA

*RRM2* rev 5′ AAATCTGCGTTGAAGCAGTGAGGC

*P53ext* fwd 5′ GAGCTGGACCTTAGGCTCCAGAAAGGACAA

*P53ex*t rev 5′ GCTGGTGTTGTTGGGCAGTGCTAGGAA

*P53int* fwd 5′ TTCTCTTTTCCTATCCTGAGTAGTGGTAA

*P53int* rev 5′ AAAGGTGATAAAAGTGAATCTGAGGCATAA

*HcoxIext* fwd 5′ GCGGTTGACTATTCTCTACAAACCACAAA

*HcoxIext* rev 5′ GGGGGTTTTATATTGATAATTGTTGTGATGAAA

*HcoxIint* fwd 5′ CGTTATCGTCACAGCCCATGCATTTGTAA

*HcoxIint* rev 5′ GAGGAGACACCTGCTAGGTGTAAGGTGAA

_______________________________________________________________________________________________*_____________________*

Compendium of primers used for PCR, qPCR and SYBR Green. Fwd: forward, rev: reverse.
